# Resting-state global brain activity induces bias in fMRI motion estimates

**DOI:** 10.1162/imag_a_00151

**Published:** 2024-05-06

**Authors:** Yixiang Mao, Conan Chen, Truong Nguyen, Thomas T. Liu

**Affiliations:** Center for Functional MRI, University of California San Diego, La Jolla, CA, United States; Departments of Electrical and Computer Engineering, University of California San Diego, La Jolla, CA, United States; Departments of Radiology, Psychiatry, and Bioengineering, University of California San Diego, La Jolla, CA, United States

**Keywords:** resting-state fMRI, multi-echo fMRI, global signal, motion parameters, resting-state functional connectivity

## Abstract

Head motion is a significant source of artifacts in resting-state fMRI (rsfMRI) studies and has been shown to affect resting-state functional connectivity (rsFC) measurements. In many rsfMRI studies, motion parameters estimated from volume registration are used to characterize head motion and to mitigate motion artifacts in rsfMRI data. While prior task-based fMRI studies have shown that task-evoked brain activations may induce temporally correlated bias in the motion estimates, resulting in artificial activations after registration, relatively little is known about neural-related bias in rsfMRI motion parameter. In this study, we demonstrate that neural-related bias exists in rsfMRI motion estimates and characterize the potential effects of the bias on rsFC estimates. Using a public multi-echo rsfMRI dataset, we use the differences between motion estimates from the first echo and second echo data as a measure of neural-induced bias. We show that the resting-state global activity of the brain, as characterized with the global signal (GS), induces bias in the motion estimates in the y- and z-translational axes. Furthermore, we demonstrate that the GS-related bias reflects superior-inferior and anterior-posterior asymmetries in the GS beta coefficient map. Finally, we demonstrate that regression with biased motion estimates can negatively bias rsFC estimates and also reduce rsFC differences between young and old subjects.

## 1 Introduction

Head motion is a major source of artifacts in fMRI data, and motion estimates from data registration are commonly used to characterize and mitigate motion-related artifacts (Dijk et al., 2012;Friston et al., 1996;Power et al., 2012,^2014^,^2015^;Satterthwaite et al., 2012;Yan et al., 2013). Prior task-based fMRI studies have demonstrated that task-evoked brain activations can induce bias in motion estimates that is temporally correlated with brain activations (Freire & Mangin, 2001;Freire et al., 2002). In contrast, relatively little is known about neural-related bias in motion estimates from resting-state fMRI (rsfMRI) data.

Detecting such bias is challenging due to the absence of ground truth head motion and neural signals in rsfMRI studies. To first order, fMRI signal changes resulting from head motion and neural activity can be distinguished based on their dependence on echo time (TE): neural activity causes TE-dependent blood-oxygen-level-dependent (BOLD) fluctuations, whereas head motion largely contributes to TE-independent non-BOLD signal changes. Taking advantage of these differences, prior studies have demonstrated that BOLD and non-BOLD signals can be distinguished using multi-echo fMRI (MEfMRI) that acquires data at different TEs (Bright & Murphy, 2013;Buur et al., 2009;Kundu et al., 2012,^2017^). Using short TE first echo data as a regressor to remove motion-related artifacts from data acquired at a longer TE with significant BOLD-weighting,Buur et al. (2009)demonstrated improvements in task-related functional activation maps (computed using the longer TE data) for individual subjects. The primary rationale behind their work was that the short TE data primarily reflected initial signal intensity*S_0_*fluctuations (due to confounds such as motion) that have minimal BOLD weighting.Bright and Murphy (2013)later demonstrated that Burr et al.’s approach could be applied to improve the accuracy of resting-state functional connectivity maps.

Burr’s findings imply that it may be feasible to identify neural-related bias by comparing motion estimates obtained from the first and second echo data. Since head motion primarily results in TE-independent signal changes, its effects should be captured in the motion estimates derived from both the first and second echo data. In contrast, there is typically minimal BOLD weighting in the first echo data but strong BOLD weighting in the second echo data. As prior work has shown that a higher level of brain activation can lead to greater bias in motion estimates (Freire & Mangin, 2001;Freire et al., 2002), potential neural-related bias should exhibit greater magnitude in the motion estimates from the second echo data as compared to those from the first echo data. Taken together, potential neural-related bias may be isolated from head motion by examining the difference between the motion estimates obtained from the first and second echo data.

Prior task-based fMRI studies have shown that brain activations with a larger spatial extent can lead to a higher level of bias in the motion estimates (Freire & Mangin, 2001;Freire et al., 2002). This finding motivates us to examine whether global brain activity in rsfMRI leads to bias in the motion estimates. In this study, we used the global signal (GS), calculated as the mean fMRI signal over the voxels within the brain, as a proxy for global brain activity. While the interpretation of the GS is still controversial (reviewed inT. T. Liu et al., 2017), there is growing evidence suggesting that the GS is linked to global neural activity (Falahpour et al., 2016,^2018^;Gu et al., 2021;X. Liu et al., 2018;Wong et al., 2013,^2016^), in addition to other components such as low frequency confounds and spin-history effects that are not related to neural activity.

In this work, we provide a mathematical rationale for how the difference in motion estimates can isolate the effects of head motion from GS-related effects. We then characterize the GS-related bias in rsfMRI motion parameters as estimated by AFNI*3dvolreg*with a public multi-echo fMRI dataset (Setton et al., 2022;Spreng et al., 2022). To reflect the GS-related effects that would be typically observed in single-echo fMRI studies, we use the second echo data, which were acquired at a commonly used TE (30 ms), for computation of the GS. Furthermore, we investigate the consequences of using biased estimates as regressors in resting-state functional connectivity (rsFC) analyses.

## 2 Methods

### 2.1 Subjects and MEfMRI data acquisition

We used a public dataset (denoted as the Cornell-York dataset and described inSetton et al. (2022)andSpreng et al. (2022))downloaded from OpenNeuro (dataset ds003592). The Cornell-York dataset includes multi-echo fMRI data collected from 301 healthy subjects (181 younger: mean age = 22.59 y, SD = 3.27 y, 57% female; and 120 older adults: mean age = 68.63 y, SD = 6.44 y, 54% female). The data from 238 subjects were acquired on a 3 T GE Discovery MR750 MRI scanner with a 32-channel head coil. The data from the remaining 63 subjects were collected on a 3 T Siemens Trio MRI scanner with a 32-channel head coil. For each subject, two 10-min resting-state runs were acquired using an ME EPI sequence on the GE scanner (204 volumes; TR = 3000 ms; TE = 13.7,30,47 ms; flip angle = 83°; FOV = 210 mm; voxel size = 3 × 3 × 3 mm^3^; matrix size = 72 × 72 × 46; SENSE acceleration factor = 2.5; phase encoding direction: A-P) or on the Siemens scanner (200 volumes; TR = 3000 ms; TE = 14,29.96,45.92 ms; flip angle = 83°; FOV = 216 mm; voxel size = 3.4 × 3.4 × 3 mm^3^; matrix size = 64 × 64 × 43; GRAPPA acceleration factor = 3; phase encoding direction: A-P). For both scan protocols, there was no acceleration in the slice direction. The subjects were instructed to stay awake and lie still with their eyes open during the scans.

### 2.2 Data preprocessing

AFNI and MATLAB were used for data preprocessing (Cox, 1996). As described in detail below and summarized in Supplementary MaterialFigure S1, we used standard AFNI algorithms for resampling (3dResample), registration (3dVolreg), mask estimation (3dAutomask), and transformation to standard space (align_epi_anat.py, auto_warp.py, and 3dNwarpApply). The rationale behind our approach was to use the minimal processing needed to demonstrate the relationship between the various metrics, such as the global signal and motion estimates. The fMRI data from the first and second echoes were used and denoted as e1 and e2, respectively. The data were first reoriented to Right-Anterior-Inferior (RAI) orientation (AFNI*3dresample -orient RAI*), and the first 6 TRs of the data were discarded to allow magnetization to reach a steady state.

### 2.3 Calculation of the motion estimates and global signal

In this study, we examined the motion estimates from AFNI*3dvolreg*(Cox & Jesmanowicz, 1999) using the first volume as the reference volume. The default weights used in registration were disabled by feeding an all-ones image to the -weight option. This approach weights all voxels equally during registration and simplifies the theory. As shown in Supplementary Material (Fig. S2), similar results were obtained when using the default weights. For each run, the motion was estimated separately for the e1 and e2 data, resulting in two sets of estimates. The motion parameters were multiplied by —1 to represent the movement of the volumes as compared to the reference volume (seeAppendix A).

For each run, the global signal (GS) was calculated from the registered e2 data. As shown in Supplementary Material (Fig. S2), nearly identical results were obtained when the GS was calculated from the unregistered e2 data. Before calculating the GS, each voxel’s signal was normalized to represent the percent BOLD signal change from the voxel’s mean signal value (computed over the duration of the scan), as the percent signal change has a meaningful connection with brain physiology (T. T. Liu et al., 2017). Then, the GS was computed by averaging over all voxels within the brain (brain masks were formed by AFNI*3dAutomask*). Finally, the mean and the linear and quadratic trends were regressed out from the motion estimates and GS, as these are typically considered to represent confounds not related to neural activity (T. T. Liu et al., 2017).

### 2.4 Identifying BOLD-weighted GS bias in the motion estimates

To characterize BOLD-weighted GS bias in the motion estimates, we considered a simple signal model for the e1 and e2 motion estimates. Let$m_{e1}\in {\mathbb{R}}^{K\times 1}$and$m_{e2}\in {\mathbb{R}}^{K\times 1}$represent the motion estimates of one motion axis each from e1 and e2, respectively, whereKis the number of volumes. We model the motion estimates as the weighted sum of head motion, BOLD-weighted GS bias, and estimation error,

$$\begin{array}{ll} m_{e1} & =\alpha_{e1}m_{h}+\beta_{e1}{\text{TE}}_{1}g_{s}+\epsilon_{e1}\\ m_{e2} & =\alpha_{e2}m_{h}+\beta_{e2}{\text{TE}}_{2}g_{s}+\epsilon_{e2} \end{array}$$ (1)

where$m_{h}\in {\mathbb{R}}^{K\times 1}$represents head motion,$\alpha_{ei}$is the regression weight corresponding to head motion for theith echo,$g_{s}\in {\mathbb{R}}^{K\times 1}$is the GS,${\text{TE}}_{1}$and${\text{TE}}_{2}$are the first and second echo times, respectively,$\beta_{ei}$is the regression weight of the BOLD-weighted GS bias for theith echo, and$\epsilon_{ei}\in {\mathbb{R}}^{K\times 1}$is the estimation error for theith echo. With the above model, subtracting$m_{e1}$from$m_{e2}$yields

$$\begin{array}{l} \Delta m=m_{e2}-m_{e1}=(\alpha_{e2}-\alpha_{e1})m_{h}\\ \;\;\;\;\;\;\;\;\;\;\;\;\;\;\;+\left(\beta_{e2}{\text{TE}}_{2}-\beta_{e1}{\text{TE}}_{1}\right)g_{s}+\epsilon_{e2}-\epsilon_{e1} \end{array}$$ (2)

Since prior findings suggest that head motion can be accurately estimated from both e1 and e2 data (Buur et al., 2009;Speck & Hennig, 2001), we assume that$\alpha_{e1}\approx \alpha_{e2}$, yielding

$$\Delta m\approx \left(\beta_{e2}{\text{TE}}_{2}-\beta_{e1}{\text{TE}}_{1}\right)g_{s}+\epsilon_{e2}-\epsilon_{e1}$$ (3)

Note thatΔmisolates BOLD-weighted GS bias from head motion. Therefore, we can examine the presence of potential bias by assessing the significance of the correlation betweenΔmand the GS. For each run and motion axis, we calculated the correlation betweenΔmand the GS (denoted asr(Δm, GS)) and assessed the statistical significance ofr(Δm, GS)values on a per-run basis using empirical null distributions. For each motion axis, a null distribution was formed by calculatingr(Δm,GS)values using all possible permutations across runs, that is, pairing the GS from one run toΔmfrom other runs and looping over all runs. The resulting null distributions consisted of 361,802 samples. Then, for each motion axis, we used the null distribution to compute the two-sided p-value associated with ther(Δm,GS)value calculated from each run’s measured data. A p-value threshold of 0.05 was divided by 602 (the number of runs) to correct for multiple comparisons, and the Bonferroni-corrected threshold was used to determine the significant correlations. Finally, we calculated the percentage of runs that show significantr(Δm,GS)values for each motion axis.

In this study, we used the GS to represent the global activity of the brain. To reduce the potential effect of motion artifacts in the GS, we repeated the above analysis after regressing out the e1 motion regressors from both the GS andΔm. The e1 motion regressors included the six motion parameters estimated from the e1 data and their first derivatives.

### 2.5 Spatial maps underlying r(Δm,GS)

To provide insight into the mechanisms underlying BOLD-weighted GS bias, we derived an empirical approximation forr(Δm,GS). As shown inAppendices BandC,

$$r\left(\Delta m,\text{GS}\right)\approx \frac{∥g_{s}∥}{∥\Delta m∥}\left(\frac{\beta_{g_{s}}^{T}{,}_{e2}d_{e2}}{∥d_{e2}{∥}^{2}}-\frac{\beta_{g_{s}}^{T}{,}_{e1}d_{e1}}{∥d_{e1}{∥}^{2}}\right)$$ (4)

where$\beta_{g_{s}}{,}_{ei}\in {\mathbb{R}}^{N\times 1}$is the GS beta coefficient map for theith echo,$d_{ei}\in {\mathbb{R}}^{N\times 1}$is the spatial derivative image with respect to (w.r.t.) one motion axis of theith echo,Nis the number of voxels, and∥⋅∥denotes theL2-norm. For each run, the GS beta coefficient map$\beta_{g_{s}}{,}_{ei}=\frac{Y_{ei}g_{s}}{∥g_{s}{∥}^{2}}$was calculated from the linear fit of the GS to the unregistered functional data$Y_{ei}\in {\mathbb{R}}^{N\times K}$of theith echo.

We calculated the spatial derivative images w.r.t. the motion axes following the algorithm implemented in AFNI*3dvolreg*. For each run and each echo, the spatial derivative images were calculated based on the reference volume used in motion estimation. Denoting the reference image of theith echo as$y_{r,ei}\in {\mathbb{R}}^{N\times 1}$, the spatial derivative image of theith echo w.r.t thejth motion axis was calculated as

$$\begin{array}{l} d_{ei,j}=\frac{T_{j}(y_{r,ei},\epsilon_{j})\;\;-\;\;T_{j}(y_{r,ei},\;\;-\;\;\epsilon_{j})}{2\epsilon_{j}},\\ \;\;\;\;\;\;\;\;\;\;\;\;\;\;\;\;i=1,2,j\in \left\{\text{Rx},\;\text{Ry},\;\text{Rz},\text{Tx},\text{Ty},\text{Tz}\right\} \end{array}$$ (5)

where$T_{j}:{\mathbb{R}}^{N\times 1}\to {\mathbb{R}}^{N\times 1}$is the function that transforms the reference volume along thejth motion axis;$\epsilon_{j}$is the transformation parameter; Rx, Ry, and Rz represent x-, y-, and z-rotation, respectively; and Tx, Ty, and Tz represent x-, y-, and z-translation, respectively. When calculating the spatial derivative images, the transformation was performed with AFNI*3drotate -heptic*. For the translational motion axes,ϵwas set to2.1mm. For the rotational motion axes,ϵwas set to$0.4^{\circ }$. Theseϵvalues were determined by AFNI*3dvolreg*based on the spatial resolution of the functional data.

### 2.6 Effect of the bias on ROI-ROI FC via motion regression

In this work, we investigated the effect of regression with biased motion estimates on rsFC estimates between nodes within the default mode network (DMN) and dorsal attention network (DAN). The regions of interest (ROIs) within these networks were defined inDijk et al. (2010), with four ROIs in the DMN (posterior cingulate cortex (PCC), lateral parietal cortex (LatPar), medial prefrontal cortex (mPFC), and Hippocampal formation (HF)) and three ROIs in the DAN (frontal eye field (FEF), intraparietal cortex (IPS), and middle temporal area (MT+)). Seed ROIs were created using a sphere with a diameter of 12 mm centered about each seed coordinate (Wong et al., 2013). The left and right ROIs were combined to form bilateral ROIs (See Supplementary MaterialFig. S3for depiction of ROIs). Prior to averaging signals within each ROI, the e2 data after volume registration were transferred to MNI space using AFNI programs align_epi_anat.py, auto_warp.py, and 3dNwarpApply. As supplementary analyses, we also considered (1) transformation to standard space using a custom young-old template (Laurita et al., 2020) and (2) rsFC analysis using a 200-region parcellation of the brain (Schaefer et al., 2018).

To reduce the confounding effects of head motion, motion censoring was performed before motion regression. For each run, framewise displacement (FD) was calculated based on six motion parameters estimated from the e1 data (the calculation of FD follows the description inPower et al. (2012)). Volumes with FD values larger than 0.2 mm were censored. We show in Supplementary Material (Figs. S7 and S8) that motion censoring has little impact on the effect of regression with biased motion estimates on rsFC.

After motion censoring, we calculated and compared the ROI-ROI FC after e2 and e1 motion regression to assess the effect of potential bias on FC analysis. For each ROI, the ROI-based seed signal was calculated by averaging the percent change BOLD signal over the voxels in that ROI. Then, the motion regressors, including the six motion parameters and their first derivatives, were regressed out from the ROI signals. For each pair of ROIs, the ROI-ROI FC was computed as the Pearson’s correlation coefficient between the ROI average signals. Correlation values were converted to z-scores using the Fisher-z transformation. Note that all statistical comparisons were performed on the z-scores. In some plots, the r-values are also presented to support the interpretation of the results. The differences in the ROI-ROI FC calculated after e2 and e1 motion regression were calculated to assess the effect of the bias on FC. The e2-e1 differences in r-values and z-scores were denoted asΔrandΔz, respectively.

For each run, the level of head motion was measured by the mean FD value calculated by averaging over the FD values across time. The level of the GS was measured by the GS amplitude (aGS) computed as the standard deviation of the GS after motion censoring. We used 2D scaled histograms to examine the distribution of theΔzvalues (averaged over all ROI pairs) as a function of aGS and motion (mean FD). Based on this examination, we then arranged the data into four groups based on aGS and mean FD levels and characterized the meanΔzvalues for each ROI pair averaged over runs within each group. All the runs were first divided into two groups based on their mean FD values. Runs with mean FD values larger than the group median were classified as high motion runs, and the remaining runs were classified as low motion runs. Then, within each FD-based group, the runs were further divided into two groups based on aGS. Runs with aGS values larger than the group median were classified as high aGS runs, and the remaining runs were classified as low aGS runs. Consequently, we formed four groups of runs: (1) low motion and high aGS runs, (2) low motion and low aGS runs, (3) high motion and high aGS runs, and (4) high motion and low aGS runs. A one-way ANOVA was calculated on meanΔzover all ROI pairs to assess whether there was a group effect. Post-hoc two-sample t-tests were calculated to characterize the differences between pairs of groups.

Additionally, to verify that the effect of the bias on FC was dominated by the motion estimates from the axes where we found GS-induced bias, we evaluated the effect of two subsets of motion regressors. One of the subsets included the motion estimates and their first derivatives from the Ty and Tz axes (where we found GS-induced bias), while the other subset included the motion regressors from the other four motion axes, where minimal GS-induced was observed.

### 2.7 Effect of the bias on young vs. old group-level FC analysis

We first examined whether the bias in motion estimates affects the ROI-ROI FC of the young and old subjects differently by comparingΔrandΔzbetween the young and old runs. Furthermore, we investigated if the bias alters the group-level FC analysis between the young and old subjects. For each pair of ROIs, the significance of the FC differences between the young and old subjects was assessed by a permutation test with$1\times 10^{7}$random permutations to allow us to apply p-value thresholds of 0.01,$1\times 10^{-3}$and$1\times 10^{-6}$. Also, the effect size of the differences was measured with Cohen’sd. The FC differences and the significance and effect size of the differences calculated after e1 and e2 motion regression were compared.

## 3 Results

### 3.1 Examples of the GS and motion estimates

**Fig. 1.**
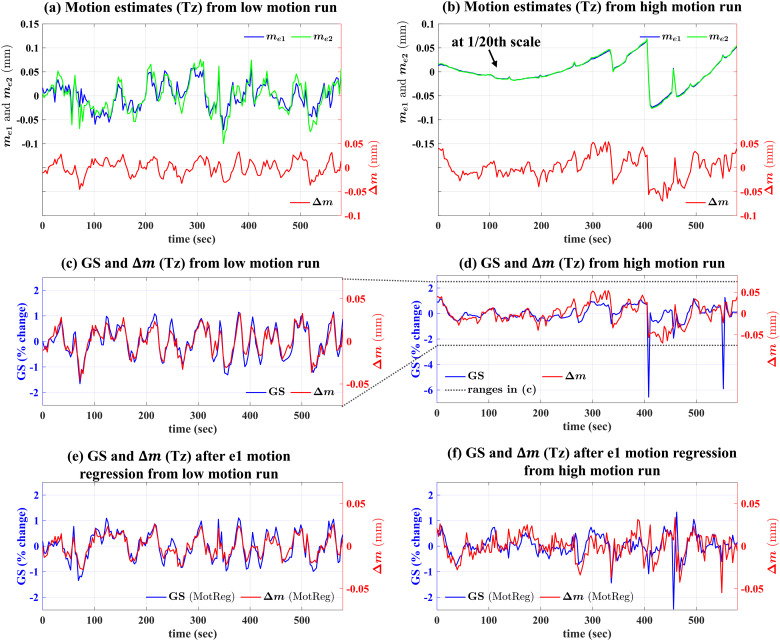
Motion estimates in the Tz axis, including$m_{e1}$(blue),$m_{e2}$(green), andΔm(red) from (a) low and (b) high motion runs. Panels (c) and (d) show the GS (blue) andΔm(red) from the low and high motion runs, respectively. Panels (e) and (f) show the GS (blue) andΔm(red) after motion regression (denoted as MotReg; with$m_{e1}$as regressor) from the low and high motion runs, respectively.

As discussed below, we found a significant association between the GS andΔmin the Tz and Ty axes. To provide a qualitative view,Figure 1(a, b) shows motion estimates in the Tz axis, including${\text{m}}_{e1}$(blue),${\text{m}}_{e2}$(green), andΔm(red) from two example runs with (a) low and (b) high levels of motion. Note that for the high motion run,$m_{e1}$and$m_{e2}$are plotted at1/20th scale to facilitate comparison with$m_{e1}$and$m_{e2}$from the low motion run. As shown in these subfigures,Δmestimates from both runs fluctuate in a similar range from−0.05to0.05mm (std(Δm)=0.017and0.025for the low and high motion runs, respectively). In contrast, the standard deviations of$m_{e1}$and$m_{e2}$from the high motion run (std$(m_{e1})=0.583$, std$(m_{e2})=0.602$) are an order of magnitude larger than the standard deviations of$m_{e1}$and$m_{e2}$from the low motion run (std$(m_{e1})=0.027$, std$(m_{e2})=0.034$).

Figure 1(c) and (d) show the GS andΔmfrom the low motion and high motion runs, respectively. For the low motion run,Δmcovaries with the GS throughout the run, leading to a strongr(Δm, GS)of 0.93. The high motion run shows a weakerr(Δm, GS)of 0.54 as compared to the low motion run, and reflects motion artifacts in the GS. After motion regression (with$m_{e1}$; panels e and f), ther(Δm, GS)for the high motion run increases to 0.62, while ther(Δm, GS)of the low motion run remains at a high value of 0.92, suggesting that the relation between the GS andΔmis enhanced when motion artifacts are minimized.

### 3.2 Significance testing for r(Δm, GS) values

**Fig. 2.**
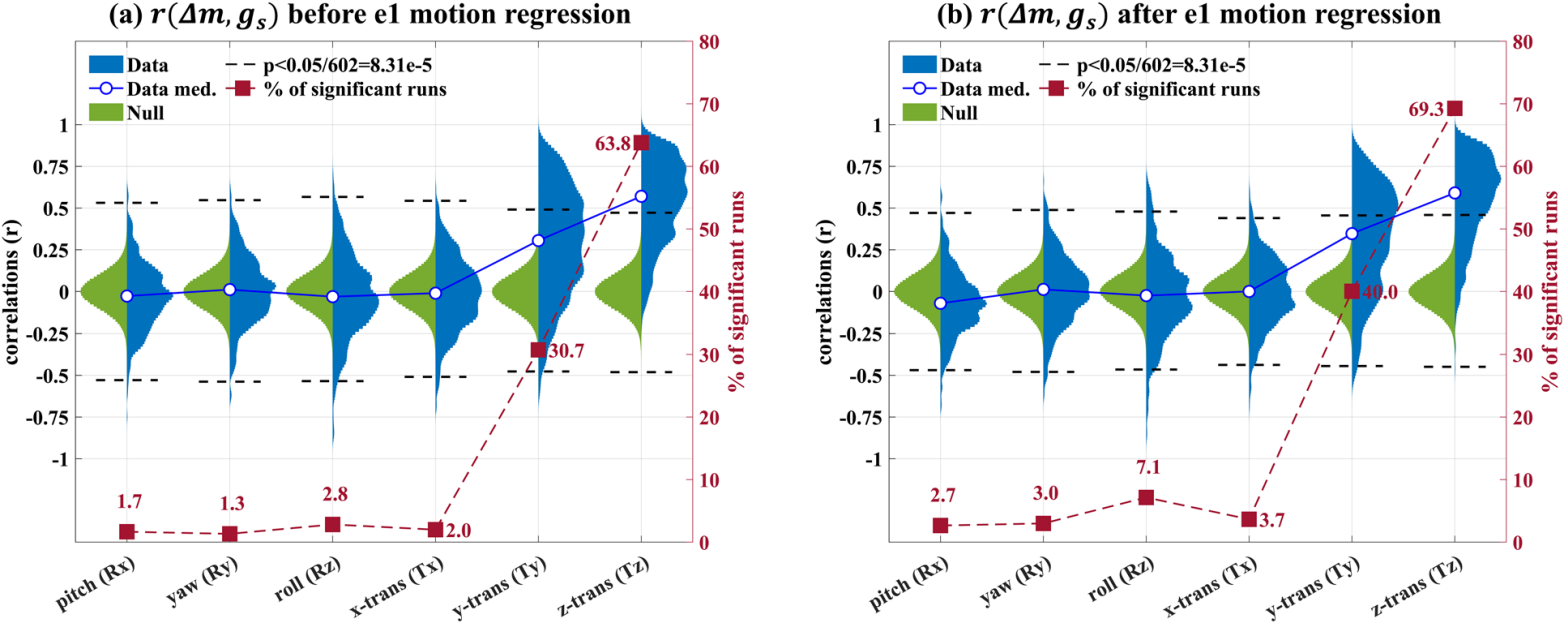
Two-sided violin plots showing the distributions ofr(Δm, GS)values (blue) and the empirical null distributions (green) for all six motion axes (a) before and (b) after e1 motion regression. The blue solid lines and circles represent the median values for each data distribution. For each motion axis, a Bonferroni-corrected run-wise p-value threshold of 0.05/602 was used, where 602 is the number of runs. The black dashed lines showr(Δm, GS)values that correspond to the p-value thresholds (two-sided) assessed from the empirical null distributions. The dark red dashed lines and square markers represent the percent of the runs with significantr(Δm, GS)values.

To examine the presence of BOLD-weighted GS bias over runs and motion axes, we assessed the significance ofr(Δm, GS)values on a per-run and per-axis basis using permutation-based empirical null distributions.Figure 2shows two-sided violin plots of the distributions ofr(Δ***m, GS)***values (blue) and the empirical null distributions (green) for all six motion axes (a) before and (b) after e1 motion regression. The blue solid lines and circles represent the median values for each distribution of measuredr(Δm, GS)values. The dashed lines represent ther(Δm, GS)values corresponding to a Bonferroni-corrected p-value threshold of 0.05 (two-sided) assessed from the empirical null distributions. The dark red square markers represent the percentages of runs showingr(Δm, GS)values that are significantly different from zero.

In the Tz axis, 63.8% and 69.3% of the runs show significant positiver(Δm, GS)values before and after e1 motion regression, respectively. The group medianr(Δm, GS)value increases from 0.57 to 0.59 after e1 motion regression, with 97 out of 602 total runs showingr(Δm, GS)values larger than 0.8 after e1 motion regression. In the Ty axis, 30.7% and 40.0% of the runs show significantr(Δm, GS)values before and after e1 motion regression, respectively. The group medianr(Δm, GS)value increases from 0.31 to 0.35 after e1 motion regression.Figures S4 and S5show examples of the GS and the motion estimates, including${\text{m}}_{e1}$,${\text{m}}_{e2},$andΔmin the Tz and Ty axes, respectively. Together, these findings indicate the presence of BOLD-weighted GS bias in the Tz and Ty motion estimates.

For the other motion axes (Rx, Ry, Rz, and Tx), the percent of significantr(Δm, GS)values fluctuates around 5%, ranging from 1.3% to 7.1%, indicating minimal BOLD-weighted GS bias for these axes. As described in Supplementary Material, a preliminary examination shows that bias is also observed when using other software packages, such as SPM (Friston et al., 1995), FSL (Jenkinson & Smith, 2001;Jenkinson et al., 2002), and ANTS (Avants et al., 2011,^2014^). The bias observed with SPM is similar to that observed with AFNI, while FSL and ANTS show relatively lower levels of bias.

### 3.3 Visualizing the spatial maps underlying r(Δm, GS) values

**Fig. 3.**
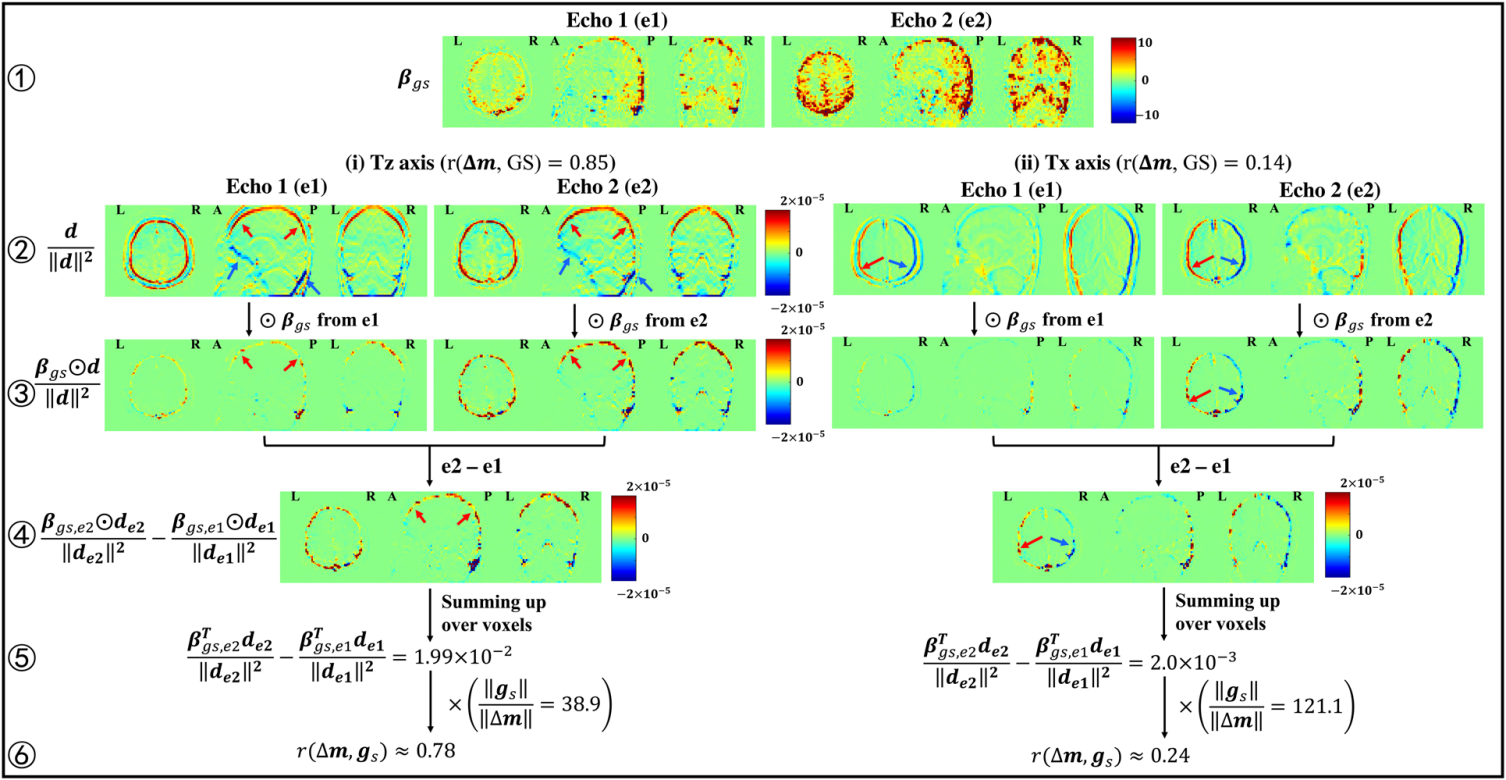
Visualization of the spatial maps underlyingr(Δm, GS)in the Tz (rows 2-6 on the left) and Tx (rows 2-6 on the right) axes from an example run. For each map, three representative slices (one axial, one sagittal, and one coronal) are plotted. From top to bottom, rows 1 through 3 show the$\beta_{{\text{g}}_{s}}$,$\frac{\text{d}}{∥\text{d}{∥}^{2}}$, and$\frac{\beta_{{\text{g}}_{s}}⊙\text{d}}{∥\text{d}{∥}^{2}}$maps from e1 and e2, where⊙represents element-wise multiplication. Row 4 shows$\frac{\beta_{{\text{g}}_{s}}⊙\text{d}}{∥\text{d}{∥}^{2}}$difference (e2–e1) maps. From left to right in rows 2 and 3, the first and second columns show the e1 and e2 maps in the Tz axis, and the third and fourth columns show the e1 and e2 maps in the Tx axis. The red and blue arrows point to brain regions showing high positive and negative values in the maps, respectively.

In the previous section, BOLD-weighted GS bias in the motion estimates was identified by examining the temporal correlations between the GS andΔm. Furthermore, as described inEq. 4,r(Δm, GS)can be approximated as the product of the difference of$\frac{\beta_{{\text{g}}_{\text{s}}}^{T}\text{d}}{∥\text{d}{∥}^{2}}$values from e2 and e1 and a scaling factor$\frac{∥{\text{g}}_{s}∥}{∥\Delta \text{m}∥}$. The approximation provides a unique angle to interpret the GS bias by looking at the relation of the$\beta_{{\text{g}}_{s}}$and***d***spatial maps.Figure 3visualizes$\beta_{{\text{g}}_{s}}$,$\frac{\text{d}}{∥\text{d}{∥}^{2}}$,$\frac{\beta_{{\text{g}}_{s}}⊙\text{d}}{∥\text{d}{∥}^{2}}$, and the$\frac{\beta_{{\text{g}}_{s}}⊙\text{d}}{∥\text{d}{∥}^{2}}$difference maps in the Tz and Tx axes from an example run, where⊙represents element-wise multiplication. Note that larger magnitudes in the GS beta coefficient$\beta_{{\text{g}}_{s}}$maps reflect a greater presence of the GS in a voxel’s time series; larger magnitudes in the normalized spatial derivative$\frac{\text{d}}{∥\text{d}{∥}^{2}}$maps indicate voxels with relatively higher spatial derivatives; larger magnitudes in the element-wise multiplication$\frac{\beta_{{\text{g}}_{s}}⊙\text{d}}{∥\text{d}{∥}^{2}}$maps indicate voxels for which both the GS beta coefficients and normalized spatial derivatives have relatively high values; and larger magnitudes in the element-wise difference maps typically indicate voxels for which the values in the element-wise map for the second echo$\frac{\beta_{{\text{g}}_{s}}{,}_{e2}⊙{\text{d}}_{e2}}{∥{\text{d}}_{e2}{∥}^{2}}$are greater than the corresponding values in the element-wise map for the first echo$\frac{\beta_{{\text{g}}_{s}}{,}_{e1}⊙{\text{d}}_{e1}}{∥{\text{d}}_{e1}{∥}^{2}}$, where the differences are driven for the most part by the greater$\beta_{{\text{g}}_{s}}$values observed for the second echo.

In the Tz axis,$\frac{\text{d}}{∥\text{d}{∥}^{2}}$exhibits high positive and negative values at the superior and inferior edges of the brain, respectively, whereas$\beta_{{\text{g}}_{s}}$exhibits positive values across the cortex, including the superior edge of the brain, but exhibits relatively low values (approaching zero) along the inferior edge. As a result, most of the high values in$\frac{\beta_{{\text{g}}_{s}}⊙\text{d}}{∥\text{d}{∥}^{2}}$are positive with greater amplitudes for e2, resulting in high positive values in the$\frac{\beta_{{\text{g}}_{s}}⊙\text{d}}{∥\text{d}{∥}^{2}}$difference map. Consequently, summing up the$\frac{\beta_{{\text{g}}_{s}}⊙\text{d}}{∥\text{d}{∥}^{2}}$difference map leads to a highr(Δm, GS)value. In contrast, for the Tx axis,$\frac{\text{d}}{∥\text{d}{∥}^{2}}$shows high positive and negative values on the left and right edges, respectively, whereas$\beta_{{\text{g}}_{s}}$shows high positive values across the cortex, including both the left and right edges. The resulting high positive and negative values observed in the$\frac{\beta_{{\text{g}}_{s}}⊙\text{d}}{∥\text{d}{∥}^{2}}$difference map tend to cancel out, resulting in a lowerr(Δm, GS)value. Together, these observations suggest that the GS bias in the Tz motion estimates results from$\beta_{{\text{g}}_{s}}$and***d***sharing a relatively similar top-bottom asymmetric spatial pattern.

In the Ty axis, as shown inFigure S6,$\frac{\text{d}}{∥\text{d}{∥}^{2}}$exhibits negative and positive values at the anterior and posterior edges of the brain, respectively, and$\beta_{{\text{g}}_{s}}$exhibits positive values across the cortex, including the posterior edge of the brain, but exhibits relatively low values (approaching zero) along the anterior edge. As a result, the positive values in the$\frac{\beta_{{\text{g}}_{s}}⊙\text{d}}{∥\text{d}{∥}^{2}}$difference map show relatively greater amplitudes as compared to the negative values, resulting in a highr(Δm, GS)value.

Taken together, the GS-induced bias in the Ty and Tz motion estimates arises because of the interaction between the roughly odd symmetry in the respective spatial derivative maps, which exhibit high positive and negative values at the edges, and the asymmetries in the respective$\beta_{{\text{g}}_{s}}$maps. If the$\beta_{{\text{g}}_{s}}$maps exhibited even symmetric values at the edges, then the odd symmetry in the spatial derivative maps would be largely preserved when multiplying by the$\beta_{{\text{g}}_{s}}$maps, such that the high positive and negative contributions would cancel out when computing the sum over the$\frac{\beta_{{\text{g}}_{s}}\text{​}⊙\text{d}}{∥\text{d}{∥}^{2}}$difference maps. However, because the$\beta_{{\text{g}}_{s}}$maps show higher values at the superior and posterior parts of the brain as compared to the inferior and anterior parts, the odd symmetry in the spatial derivative maps is not preserved and the high positive and negative contributions do not cancel out when computing the sum of the$\frac{\beta_{{\text{g}}_{s}}⊙\text{d}}{∥\text{d}{∥}^{2}}$difference maps.

As shown in Supplementary Material, the superior-inferior and posterior-anterior asymmetric spatial patterns in the$\beta_{{\text{g}}_{s}}$maps show a significant spatial correlation with the patterns observed in the vigilance template, suggesting that the asymmetric patterns may partly reflect spatial variations in the effect of vigilance variations on the fMRI signal. Furthermore, as described in Supplementary Material, image intensity variations due to factors such as inhomogeneities in the receive coil sensitivity patterns (Belaroussi et al., 2006) are another potential source of the posterior-anterior asymmetric pattern in$\beta_{{\text{g}}_{s}}$.

### 3.4 Effect of the bias on ROI-ROI FC via motion regression

In this section, we investigate how the presence of the GS-induced bias in the motion estimates affects the ROI-ROI FC by examining the differences in the ROI-ROI FC calculated after e2 and e1 motion regression. The e2–e1 differences in r-values and z-scores are denoted asΔrandΔz, respectively.

**Fig. 4.**
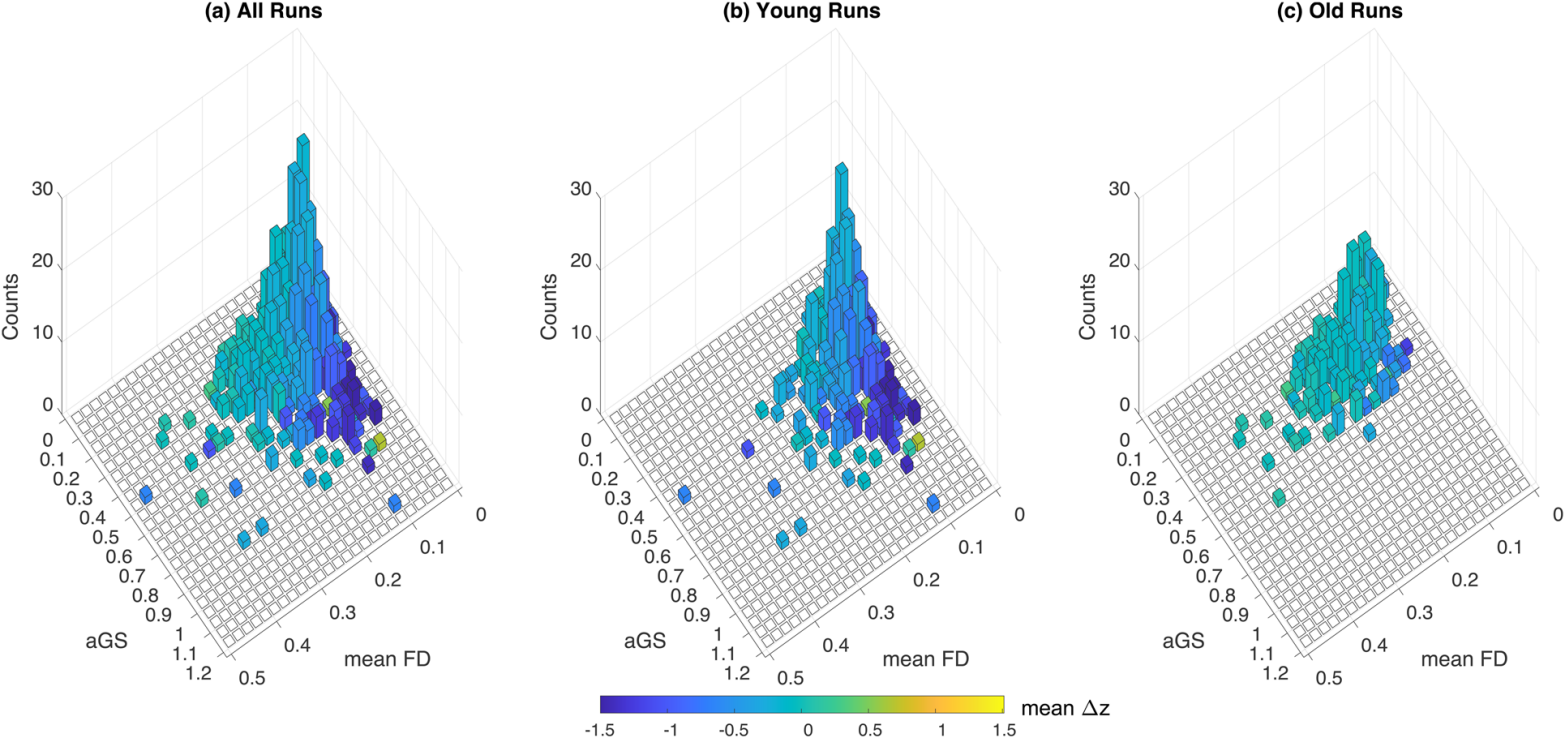
2D scaled histograms showing differences (e2–e1) in mean ROI-ROI FC (z-scores) as a function of aGS and mean FD (mm), where the heights of the bars indicate the count and the color indicates the meanΔzvalues.

Figure 4shows the distribution of meanΔzvalues (averaged over all ROI pairs) as a function of aGS and mean FD. When all runs are considered together (panel a), the most negative (dark blue)Δzvalues occur for runs with high aGS and low mean FD. This behavior is largely driven by the distribution of values for the younger subjects (panel b), which is in contrast to the distribution of values for the older subjects (panel c), where there are relatively few samples at high aGS and low mean FD. The age-related difference in the distributions gives rise to the group analysis effects addressed inSection 3.4.1. As shown inFigure S9, similar distributions are observed when considering only those runs acquired on the GE scanner.

**Fig. 5.**
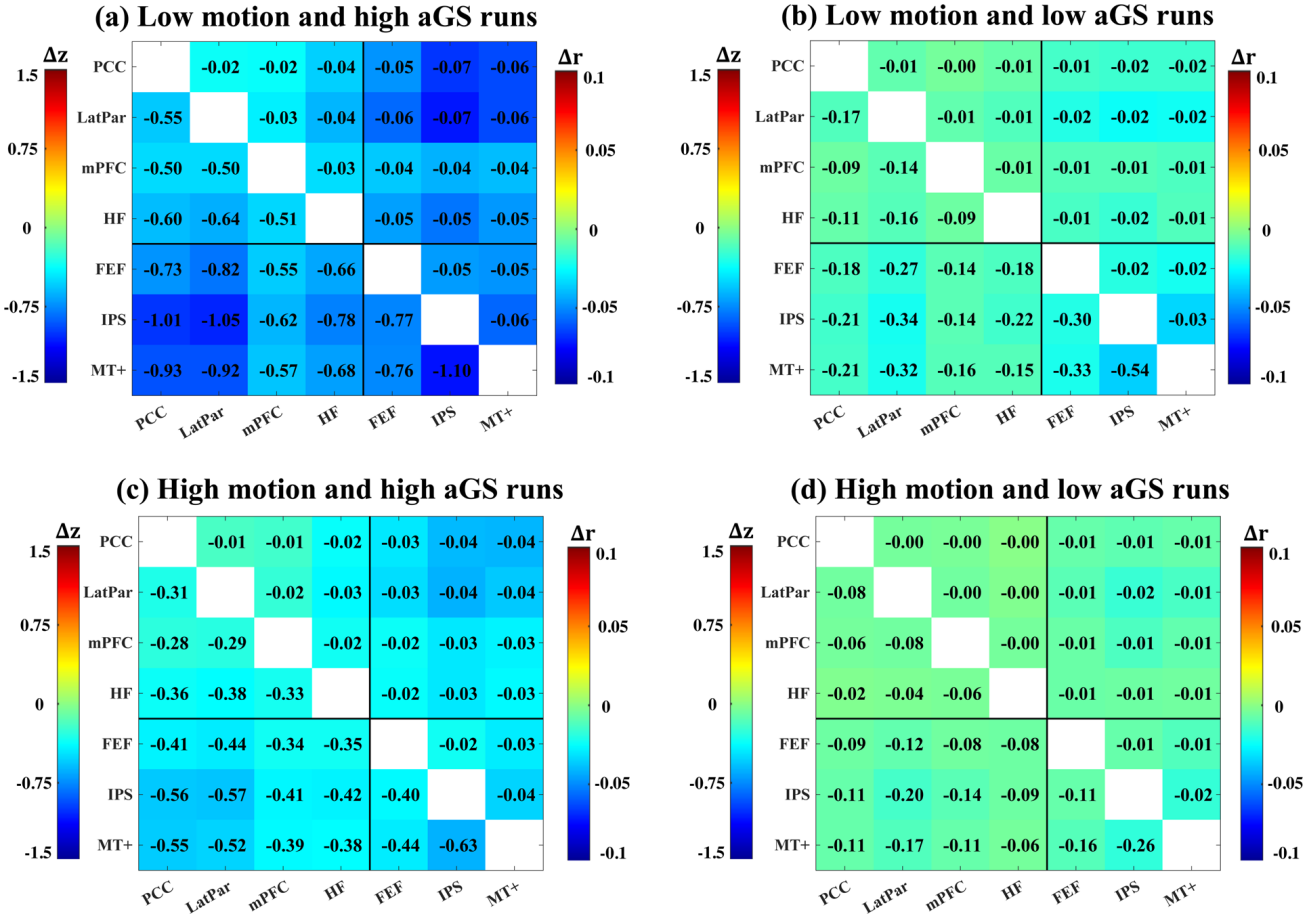
Differences (e2–e1) in ROI-ROI FC calculated after e2 and e1 motion regression. The differences were averaged across runs within each of four groups: (a) low motion and high aGS runs, (b) low motion and low aGS runs, (c) high motion and high aGS runs, and (d) high motion and low aGS runs. Each subplot is divided into an upper right triangle showing the average differences in r-values and a lower left triangle showing the average differences in z-scores.

Figure 5showsΔrandΔzfor all pairs of ROIs averaged over four groups of runs: (a) low motion and high aGS runs, (b) low motion and low aGS runs, (c) high motion and high aGS runs, and (d) high motion and low aGS runs. All four groups demonstrate negativeΔrandΔzvalues for all pairs of ROIs, suggesting that the bias in the motion estimates can reduce FC estimates via motion regression. Comparing groups, we observed that the runs with low motion and high aGS exhibit the most negativeΔrandΔzvalues (Δrranging from -0.02 to -0.07 andΔzranging from -0.50 to -1.10), while the runs with high motion and low aGS showΔrandΔzvalues close to zero (Δrranging from -0.00 to -0.02 andΔzranging from -0.02 to -0.26).

Furthermore, a one-way ANOVA on the meanΔzvalues calculated over ROI pairs showed that there is a significant ($p<1\times 10^{-6}$,$F_{3,598}=46.27$) difference among groups. The results from the post-hoc t-tests indicate that the low motion and high aGS runs exhibit significantly ($p<1\times 10^{-4}$) more negative meanΔzvalues as compared to the other three groups, whereas the high motion and low aGS runs exhibit significantly ($p<1\times 10^{-3}$) less negative meanΔzvalues as compared to the other three groups. Also, the group of low motion and low aGS runs shows a significantly ($p=6.5\times 10^{-6}$) less negative meanΔzvalue as compared to the group of high motion and high aGS runs. The distributions of the meanΔzvalues over ROI pairs for the four groups are shown inFigure S8.

Figures S10 and S11demonstrate that the observed effect of the bias is dominated by the motion estimates in the Ty and Tz axes. InFigure S10, when including only the Ty and Tz motion regressors, the results are similar to those shown inFigure 5. In contrast, when excluding the Ty and Tz motion regressors (Fig. S11), we observed minimal differences between the FC estimates obtained with e1 and e2 motion regression. Together, these results indicate that regressing out the motion estimates with GS-induced bias may lead to reductions in FC estimates, with a stronger effect for runs with higher aGS and lower head motion.

#### 3.4.1 Effect of the bias on young vs. old group-level analysis

**Fig. 6.**
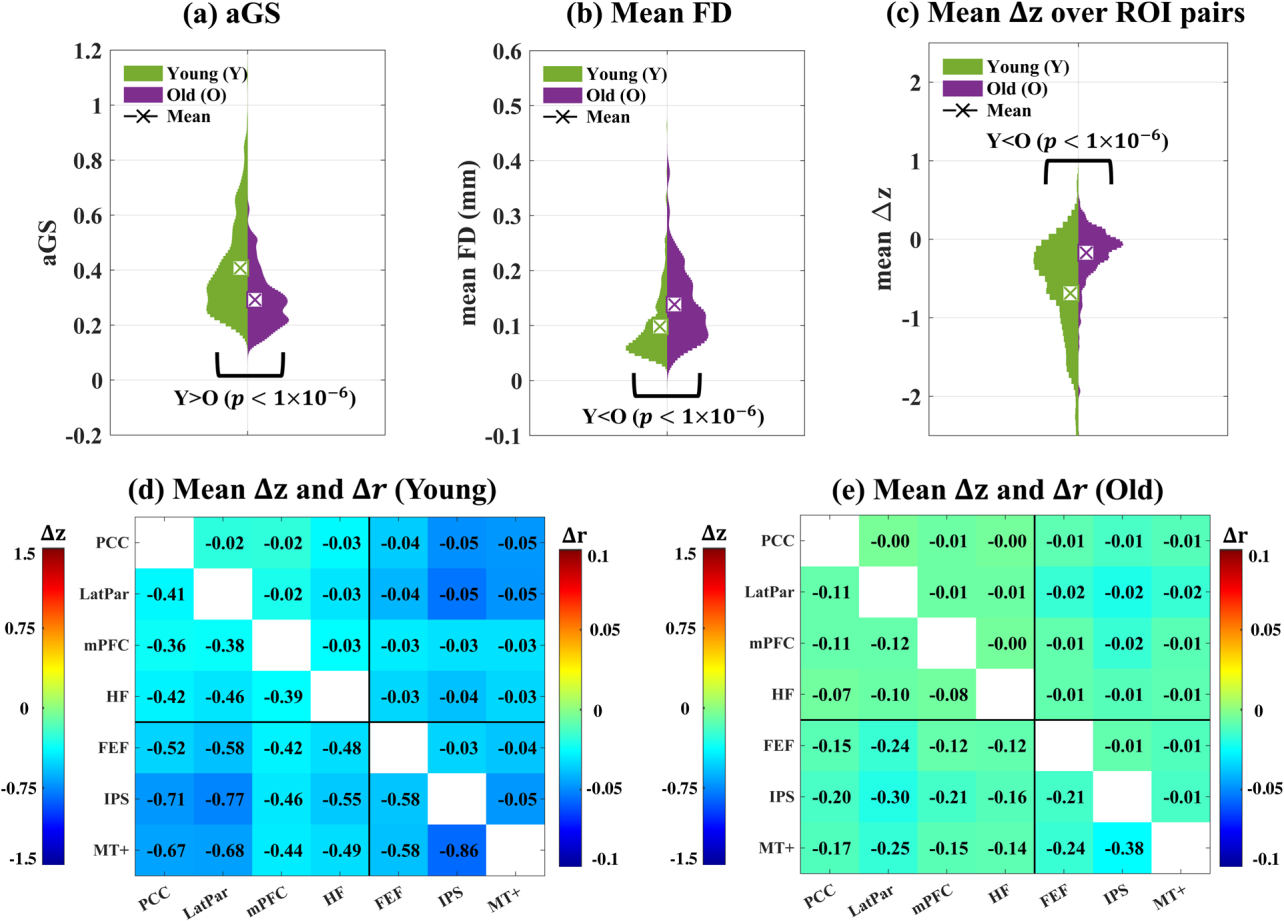
(a-c) Two-sided violin plots showing the distributions of (a) aGS, (b) mean FD, and (c) meanΔz(e2-e1) over ROI pairs for the young (green) and old (purple) subjects. Two-sided permutation tests were calculated to assess the significance thresholds for the differences between the young and old subjects. Young subjects show significantly ($p<1\times 10^{-6}$) larger aGS, smaller mean FD, and more negative meanΔzas compared to the old subjects. Panels (d-e) show the meanΔzandΔrover (d) young and (e) old subjects for all ROI pairs. Each subplot is divided into an upper right triangle showing the average differences in r-values and a lower left triangle showing the average differences in z-scores.

As shown inFigure 6 (a) and (b), young subjects show significantly ($p<1\times 10^{-6}$) higher aGS and lower mean FD values as compared to the old subjects, with the higher aGS values in the young consistent with the higher correlation values seen in the young GS topography maps presented in Supplementary MaterialFigure S12. Consequently, as shown inFigure 6 (c), the young subjects show significantly ($p<1\times 10^{-6}$) more negative meanΔz(e2-e1) values over ROI pairs as compared to the old subjects. The meanΔzvalues over runs and ROI pairs are -0.53 and -0.11 for the young and old subjects, respectively. Furthermore,Figure 6(d) and (e) visualize the meanΔrandΔzvalues over the young and old subjects for all ROI pairs, with the underlying z-score and r-value maps shown in Supplementary MaterialFigure S13(in addition, maps with global signal regression are shown inFig. S14). TheΔzvalues were only weakly correlated with the z-score differences for e1 motion regression versus no motion regression, accounting for6.8%and2.0%of the variance in the young and old groups, respectively. We observed that for all ROI pairs, the young subjects show more negativeΔrandΔzvalues as compared to the old subjects. SinceΔzandΔrreflect the effect of the bias when performing motion regression, these results suggest a greater impact of the bias on the young subjects as compared to the old subjects. Similar age-dependent reductions inΔzandΔrare observed across multiple brain networks, as shown in Supplementary MaterialFigure S15for a 200-region parcellation of the brain.

**Fig. 7.**
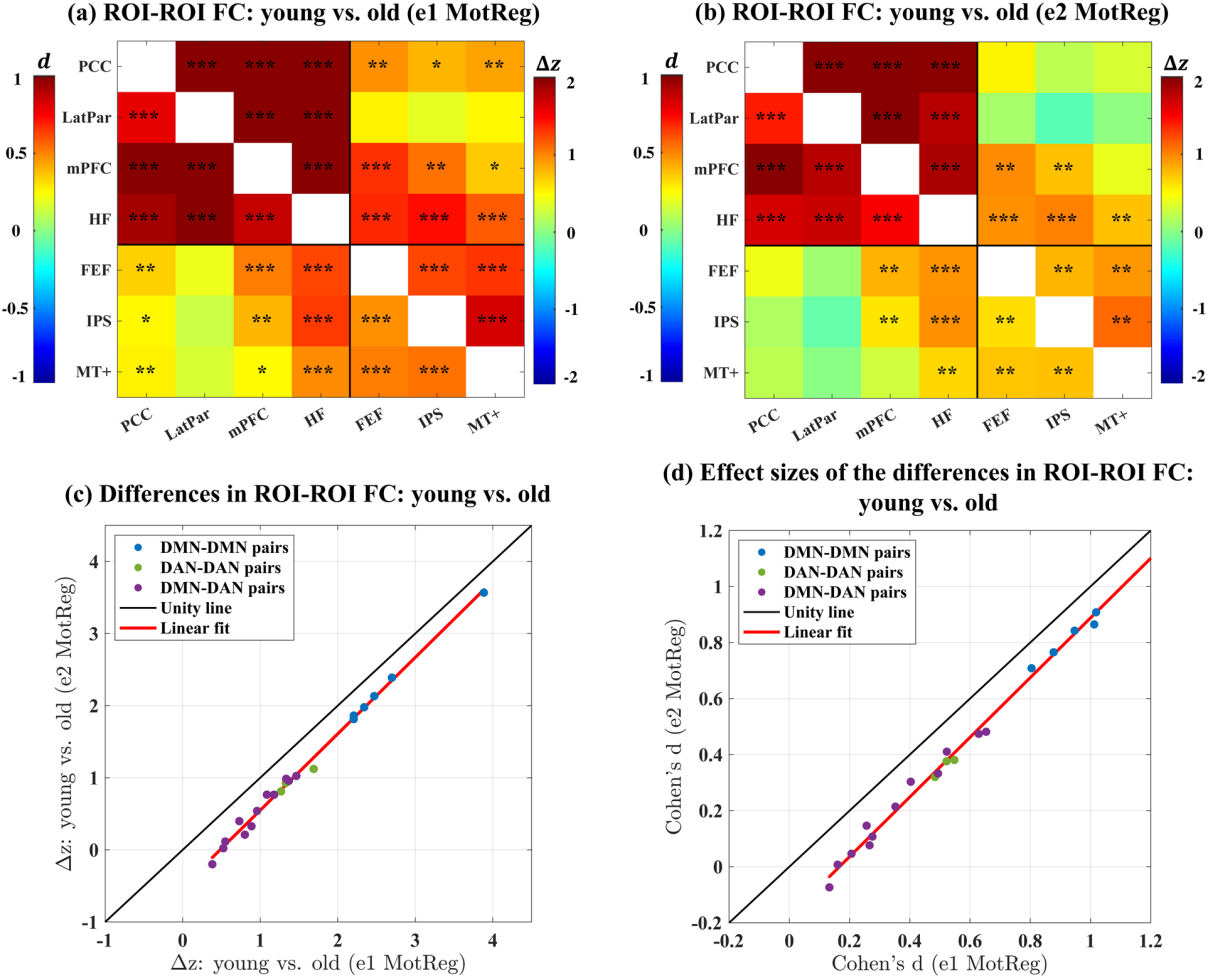
(a-b) ROI-ROI FC differences (young-old) between young and old subjects calculated after (a) e1 and (b) e2 motion regression. Each subplot is divided into an upper right triangle showing the averaged differences in z-scores between young and old subjects and a lower left triangle showing the effect size of the differences. The black asterisks indicate the statistical significance of the differences in z-scores assessed by permutation tests (*:p<0.01, **:$p<1\times 10^{-3}$, ***:$p<1\times 10^{-6}$). A positive value (red color) indicates that the young subjects show higher connectivity as compared to the old subjects. (c) Scatter plot comparing the differences in ROI-ROI FC between the young and the old subjects calculated after e1 and e2 motion regression (denoted as e1 MotReg and e2 MotReg, respectively) for all pairs of ROIs. (d) Scatter plot comparing the effect sizes of the differences in ROI-ROI FC between the young and the old subjects calculated after e1 and e2 motion regression for all pairs of ROIs.

Moreover, we examined whether the bias affects the group-level analysis between the young and the old subjects.Figure 7(a) shows the differences in ROI-ROI FC between the young and old subjects calculated after motion censoring and e1 motion regression. We observed that the young subjects show significantly (p<0.01) higher FC than the old subjects for all ROI pairs, except for the pairs between LatPar and the DAN ROIs (i.e., FEF, IPS, and MT+). However, after e2 motion regression, as shown inFigure 7(b), the significant differences for four pairs of ROIs become insignificant (p>0.01), including the pairs between (1) PCC and FEF, (2) PCC and IPS, (3) PCC and MT+, and (4) mPFC and MT+. Additionally,Figure 7(c) and (d) show that e2 motion regression reduces the FC differences between young and old for all pairs of ROIs as compared to e1 motion regression. A supplementary analysis using a custom young-old template for transformation to standard space (Laurita et al., 2020) yielded similar results, as shown inFigure S16. Furthermore, as shown inFigures S17 and S18, we verified that the observed effect is dominated by regressing out the Ty and Tz motion regressors. In addition, a supplementary analysis using an expanded model with FD as a confound yielded similar reductions in young-old FC differences with e2 motion regression, as shown inFigure S19. Together, these results demonstrate that motion regression with the e2 motion estimates can lead to a reduction in FC differences between the young and old subjects as compared to regression with the e1 motion estimates. Because both the e1 and e2 motion estimates carry information related to actual motion, the differences most likely reflect the effect of additional information carried in the e2 motion estimates.

## 4 Discussion

In this study, we used a public resting-state MEfMRI dataset to examine whether global brain activity can lead to bias in rsfMRI motion estimates and to characterize the potential impact on rsFC estimates. By examining the correlation between the GS and the difference in the motion estimates from the first and second echoes, we found evidence for GS-related bias in the Tz and Ty motion estimates. We also demonstrated that the GS-induced bias can lead to underestimation of rsFC estimates when using motion regression, with low motion and high aGS runs exhibiting the greatest reductions in FC due to the bias and high motion and low aGS runs showing minimal reductions in FC. Finally, we showed that regression with biased motion estimates can reduce rsFC differences between groups of young and old subjects, due in part to different levels of aGS and head motion between the groups.

Extending prior studies that examined bias in task-based fMRI motion estimates, we identified BOLD-weighted bias in rsfMRI motion estimates and investigated its effect on rsFC. Moreover, utilizing multi-echo fMRI data, we proposed a novel method to detect the BOLD-weighted bias in real motion estimates over a large sample of runs with rigorous statistical tests. Furthermore, to investigate the underlying cause of the observed bias, we proposed an empirical approximation tor(Δm, GS)and used this approximation to demonstrate how the presence of the GS-induced bias in the motion estimates may be attributed to the superior-inferior and posterior-anterior asymmetric spatial patterns in the GS beta coefficient map$\beta_{{\text{g}}_{s}}$. In prior work (Freire & Mangin, 2001;Freire et al., 2002), the existence of the bias in task-based fMRI was primarily illustrated using simulations and the use of experimental data was limited to one scan.

While the interpretation of the GS is still controversial (reviewed inT. T. Liu et al., 2017), there is growing evidence suggesting that the GS is linked to vigilance (also known as arousal level) (Falahpour et al., 2016,^2018^;X. Liu et al., 2018;Wong et al., 2013,^2016^) and that arousal-related GS peaks are related to changes in head motion parameters (Gu et al., 2020). For example,Falahpour et al. (2016)found that the GS is negatively correlated with EEG measures of vigilance. Furthermore, studies have shown that a vigilance template, calculated from voxel-wise correlations between the EEG vigilance measures and the fMRI signal, can be used to estimate vigilance fluctuations in fMRI scans (Chang et al., 2016;Falahpour et al., 2018;Goodale et al., 2021). As noted inSection 3.3, the presence of a significant spatial correlation between the vigilance template and the$\beta_{{\text{g}}_{s}}$maps suggests that the asymmetric spatial patterns in the$\beta_{{\text{g}}_{s}}$maps may partly reflect vigilance effects.

In this work, we performed a detailed analysis of the bias in motion estimates obtained with AFNI*3dvolreg*, which is widely used for preprocessing of fMRI data. As noted inSection 3.2, a preliminary examination indicates that lower levels of bias may be achieved with other software packages such as FSL and ANTS. Future work is needed to thoroughly examine how the choice of algorithm (and associated cost functions) affects the level of the bias in the motion estimates and to determine whether migrating to alternate algorithms constitutes an effective mitigation strategy. For example, such an effort could examine performance across the range of cost functions in the AFNI 3dAllineate program. In addition, future work focused on developing registration algorithms with reduced bias would be of interest.

In this study, we demonstrated that regression with biased motion estimates can reduce differences in rsFC between the young and old subjects, as assessed with ROIs in the DMN and DAN. Importantly, we showed that the significant rsFC differences for four pairs of ROIs became insignificant (p>0.01) due to regression with the biased motion estimates, with all four pairs corresponding to connections between the DMN and DAN. These findings suggest that regression with biased motion estimates may impede the detection of rsFC differences between the young and old groups. Because rsFC of the DMN plays a key role in understanding the aging brain (reviewed inFerreira & Busatto (2013)), it can be important for future aging studies to minimize the effect of the bias on rsFC analyses. Based on our results, investigators who are analyzing single-echo fMRI studies may want to compare findings obtained with and without Ty and Tz motion regressors. In addition, as discussed below, the impact of the biased motion estimates will be lessened when other GS-related regressors are used in the processing. When comparing results from existing aging studies with different processing methodologies, the potential effect of the bias also needs to be considered. Concerning this matter, future work that further examines the potential effect of the bias on the young versus old differences in rsFC measures would be of interest.

Our findings revealed that the effect of the bias on the young versus old rsFC analysis may be caused by different levels of aGS and head motion between the groups, with the old subjects showing a lower level of aGS and a higher level of head motion than the young subjects. The higher levels of motion in the old group have been consistently reported in prior rsfMRI studies (Hausman et al., 2022;Saccà et al., 2021), which reflects declines in executive functioning with aging (Hausman et al., 2022). A direct comparison of aGS between young and old groups does not appear to have been addressed in prior studies. However, previous studies have reported that old subjects show lower rsFC and BOLD variability (measured as the standard deviation of BOLD timeseries) in large-scale brain networks, including the DMN, compared to young subjects (Ferreira & Busatto, 2013;Grady & Garrett, 2018;Kumral et al., 2020;Nomi et al., 2017), supporting the observed lower aGS in the old subjects.

## 5 Conclusion

In conclusion, we found that resting-state global brain activity can lead to bias in Ty and Tz motion estimates obtained with a widely used motion correction algorithm. Furthermore, the bias in the motion estimates can lead to reductions in the rsFC measures obtained after motion regression, with an increasing level of bias for runs showing higher global signal amplitude and smaller head motion levels. This GS-related decrease in rsFC values is similar to the reduction seen with global signal regression (T. T. Liu et al., 2017), and therefore concerns about the effects of global signal regression may also apply when regressing out motion parameters estimated from rsfMRI data acquired at typical echo times (e.g., 30 ms). Moreover, our results show that regression with biased motion estimates can reduce group-level rsFC differences between young and old subjects. Similar effects may also be present in other rsfMRI studies in which the groups exhibit different mean levels of global signal amplitude. For rsFC studies in which the processing includes additional GS-related nuisance regressors, such as white matter and cerebrospinal fluid signals and their temporal derivatives, the potential bias due to motion regression is likely to be shared among the various GS-related regressors, especially when partial voluming effects are not adequately minimized (Behzadi et al., 2007;T. T. Liu et al., 2017). As a result when using motion regression together with either global signal regression or approaches such as CompCor (Behzadi et al., 2007), the unique contribution of the bias in the motion estimates is likely to be attenuated. On the other hand, for studies that make use of rsfMRI global activity to estimate vigilance levels (T. T. Liu & Falahpour, 2020), the potential bias in the motion estimates must be taken into account as it can affect the estimation of the metrics. Some studies, such as multi-echo fMRI studies that do not employ motion regression and instead use ICA-based approaches to remove motion-related artifacts (Setton et al., 2022), are unlikely to be affected by the bias. Overall, our results suggest that a greater degree of caution should be used when interpreting rsfMRI metrics obtained when motion regression is used in the processing of the data.

## Supplementary Material

Supplementary Material

## Data Availability

The dataset can be downloaded from openNeuro (dataset ds003592). Analysis code and files to generate the figures and results presented in this paper will be made available upon publication through the Open Science Framework DOI 10.17605/OSF.IO/SH79V.

## Appendix A Motion estimation in AFNI *3dvolreg*

In this section, we review the theory and implementation of AFNI*3dvolreg*. Let$\text{Y}=\left[{\text{y}}_{1},{\text{y}}_{2},...,{\text{y}}_{K}\right]\in {\mathbb{R}}^{N\times K}$be the functional data, whereNis the number of voxels,Kis the number of volumes, and${\text{y}}_{k}\in {\mathbb{R}}^{N\times 1}$is thekth column ofY, that is, thekth volume of the data withk=1,2,...,K. Let${\text{y}}_{r}\in {\mathbb{R}}^{N\times 1}$be the reference image used in the registration. To align${\text{y}}_{k}$to${\text{y}}_{r}$, the algorithm first estimates the motion parameters${\text{a}}_{k}\in {\mathbb{R}}^{6\times 1}$(consisting of 3 rotational and 3 translational motion parameters) by iteratively minimizing the weighted least square cost function:

$${\text{a}}_{k}=\underset{\text{a}}{\text{arg min}}||{\text{W}}^{\frac{1}{2}}\left({\text{y}}_{k}-T({\text{y}}_{r},\text{a})\right){||}_{2}^{2}$$
(A. 1)

whereWis a diagonal weight matrix, and$T:{\mathbb{R}}^{N\times 1}\to {\mathbb{R}}^{N\times 1}$is the function that performs rigid body transformation. Because we disabled the weights, we haveW=I, whereIis the identity matrix. After obtaining${\text{a}}_{k}$, the algorithm then transforms${\text{y}}_{k}$with$-{\text{a}}_{k}$, which can be written as$T\left({\text{y}}_{k},-{\text{a}}_{k}\right)$, to register${\text{y}}_{k}$to${\text{y}}_{r}$. To solve the minimization problem inEq. A.1, the algorithm linearizes the rigid body transformation with a first-order Taylor approximation, which can be written as

$$T(y_{r},a)\approx y_{r}+\sum_{j=1}^{6}a_{j}\frac{\partial T(y_{r},a)}{\partial a_{j}}$$
(A. 2)

where$a_{j}$is thejth element ofa. Furthermore, the partial derivatives inEq. A.2are approximated with finite differences:

$${\text{d}}_{j}≜\frac{\partial T\left({\text{y}}_{r},\text{a}\right)}{\partial a_{i}}\approx \frac{T\left({\text{y}}_{r},\epsilon {\text{s}}_{j}\right)-T\left({\text{y}}_{r},-\epsilon {\text{s}}_{j}\right)}{2\epsilon },j=1,\;2,...,6$$
(A. 3)

whereϵis a scalar with small magnitude and${\text{s}}_{j}$is a6×1vector whosejth element is one while the other elements are zeros. Here, we use${\text{d}}_{j}\in {\mathbb{R}}^{N\times 1}$to represent the partial derivative with respect to (w.r.t.)$a_{j}$and refer to it as the spatial derivative image w.r.t. thejth motion axis. The linearization of the transformation can then be written as

$$T\left({\text{y}}_{r},\text{a}\right)\approx {\text{y}}_{r}+\text{Da}$$
(A. 4)

where$\text{D}=\left[{\text{d}}_{1},{\text{d}}_{2},...,{\text{d}}_{6}\right]\in {\mathbb{R}}^{N\times 6}$. With the above linearization andW=I, the minimization problem inEq. A.1can be written as

$${\text{a}}_{k}=\underset{\text{a}}{\text{arg min}}||{\text{y}}_{k}\;-{\text{y}}_{r}\;-\text{Da}{||}_{2}^{2}$$
(A. 5)

In AFNI*3dvolreg*, the motion parameters are estimated by iteratively minimizing the cost function inEq. A.5. Let${\text{a}}_{k,n}$denote the motion parameters estimated at thenth iteration,

$${\text{a}}_{k,n}={\left({\text{D}}^{T}\text{D}\right)}^{-1}{\text{D}}^{T}\left({\text{y}}_{k,n}-{\text{y}}_{r}\right)\;\;\;\;n=1,2,...,N_{k}$$
(A. 6)

where$N_{k}$is the total number of iterations for thekth volume, and${\text{y}}_{k,n}$can be written as

$${\text{y}}_{k,n}=\{\begin{array}{cc} {\text{y}}_{k}, & n=1\\ T\left({\text{y}}_{k,n-1},-{\text{a}}_{k,n-1}\right), & n=2,3,...,N_{k} \end{array}$$
(A. 7)

The algorithm stops the iterative process when${\text{a}}_{k,n}$is smaller than a fixed threshold (for the dataset used in this study, the translational and rotational thresholds are0.03mm and0.02degrees, respectively) or$N_{k}$exceeds the maximum number of iterations, denoted as$N_{max}$. The algorithm uses this iterative process to deal with the approximation error in the linearization of the rigid transformation described inEq. A.4. In practice, for most volumes, the iterative process stops at the second iteration, suggesting that the parameters estimated in the second iteration are smaller than the thresholds andEq. A.4is a valid approximation. After the iterative estimation process stops, the motion parameters of thekth volume are calculated by summing the estimated parameters over all iterations:

$$a_{k}=\sum_{n=1}^{N_{k}}a_{k,n}$$
(A. 8)

Stacking the motion parameters over volumes yields

$$\text{M}=\left[{\text{a}}_{1}^{T};{\text{a}}_{2}^{T};...;{\text{a}}_{K}^{T}\right]$$
(A. 9)

Here, thejth column ofMrepresents the time series of the motion parameters of thejth motion axis (denoted as${\text{m}}_{j}$). Furthermore, in order to explicitly show the relation betweenMand the functional dataY, we define${\text{y}}_{k,n}={\text{y}}_{r}$for$N_{k}<n\leq N_{max}$so that${\text{a}}_{k,n}=0$for$N_{k}<n\leq N_{max}$. Therefore, the motion parameters can be written as

$$\begin{array}{l} M=[\sum_{n=1}^{N_{\max}}a_{1,n}^{T};\sum_{n=1}^{N_{\max}}a_{2,n};\ldots ;\sum_{n=1}^{N_{\max}}a_{K,n}^{T}]\\ \;\;\;\;\;\;=\;\;\sum_{n=1}^{N_{\max}}[{\left(y_{1,n}-y_{r}\right)}^{T};{\left(y_{2,n}-y_{r}\right)}^{T};\ldots ;{\left(y_{K,n}-y_{r}\right)}^{T}]D{\left(D^{T}D\right)}^{-1}\\ \;\;\;\;\;\;=\sum_{n=1}^{N_{\max}}(Y_{n}^{T}-1_{K}y_{r}^{T})D{\left(D^{T}D\right)}^{-1} \end{array}$$
(A. 10)

where${\text{Y}}_{n}=\left[{\text{y}}_{1,n},{\text{y}}_{2,n},...,{\text{y}}_{K,n}\right]$and$1_{K}$is aKby1all-one vector. Here,$\left({\text{Y}}_{n}^{T}-1_{K}{\text{y}}_{r}^{T}\right)\text{D}{\left({\text{D}}^{T}\text{D}\right)}^{-1}$represents the parameters estimated at thenth iteration.

In practice, the algorithm directly estimates and outputs the transformation parameters used to align${\text{y}}_{k}$to${\text{y}}_{r}$(i.e.,$-{\text{a}}_{k}$) by using−D. Therefore, in this work, the transformation parameters generated by AFNI*3dvolreg*were multiplied by−1to represent the motion parameters${\text{a}}_{k}$.

## Appendix B. Empirical approximations to the motion estimates

To explore the key terms underlyingr(Δm,GS), we derived the following empirical approximations of the motion estimates:

$$\begin{array}{l} M=\underset{\text{1st}\;\text{iteration}}{\underset{︸}{(Y^{T}-1_{K}y_{r}^{T})D{\left(D^{T}D\right)}^{-1}}}+\underset{\text{other}\;\text{iterations}}{\underset{︸}{\sum_{n=2}^{N_{\max}}(Y_{n}^{T}-1_{K}y_{r}^{T})D{\left(D^{T}D\right)}^{-1}}}\\ \;\;\;\;\;\approx M_{A1}=(Y^{T}-1_{K}y_{r}^{T})D{\left(D^{T}D\right)}^{-1}\;\;\;\;\;\;\;\;\;\;\;\;\;\;\;\;\;\;\;\;\;\;\;\;\;\;\;\;\;\;(\text{Approx}.\;1)\\ \;\;\;\;\;\approx M_{A2}=Y^{T}D{\left(D^{T}D\right)}^{-1}\;\;\;\;\;\;\;\;\;\;\;\;\;\;\;\;\;\;\;\;\;\;\;\;\;\;\;\;\;\;\;\;\;\;\;\;\;\;\;\;\;\;\;\;\;\;\;\;\;\;\;\;(\text{Approx}.\;2)\\ \;\;\;\;\;\approx M_{A3}=Y^{T}D{\left(diag(diag(D^{T}D))\right)}^{-1}\;\;\;\;\;\;\;\;\;\;\;\;\;\;\;\;\;\;\;\;\;\;\;(\text{Approx}.\;3) \end{array}$$
(B. 1)

where$1_{K}\in {\mathbb{R}}^{K\times 1}$is an all-one vector and*diag()*denotes the Matlab function diag(). The first approximation of the motion estimates, denoted as${\text{M}}_{A1}$, represents the motion parameters estimated at the first iteration and reflects the empirical observation noted above that in practice the iteration process typically stops after the first iteration. Then, because the reference image is almost orthogonal to the derivative images (i.e.,${\text{y}}_{r}^{T}\text{D}\approx 0$), we can make the second approximation, denoted as${\text{M}}_{A2}$, which represents the motion parameters estimated without subtracting the reference image from the functional data. The third approximation of the motion estimates, denoted as${\text{M}}_{A3}$, represents the motion parameters estimated after zeroing out the off-diagonal terms in${\text{D}}^{T}\text{D}$, reflecting the fact that the covariance between the derivative images in different axes is relatively small compared to the variance of the derivative images. To evaluate the approximations, the temporal correlations betweenMand the approximated motion estimates were calculated for each motion axis. As shown inFigure S23,Mis highly correlated with${\text{M}}_{A1}$(meanr=0.99),${\text{M}}_{A2}$(meanr=0.99), and${\text{M}}_{A3}$(meanr=0.91)).Figure S24displays the motion estimates before and after the approximations in the Tz and Ty axes from three example runs.

With the third approximation, the motion estimates of one motion axis, denoted as${\text{m}}_{A3}\in {\mathbb{R}}^{K\times 1},$can be written as

$${\text{m}}_{A3}=\frac{{\text{Y}}^{T}\text{d}}{∥\text{d}{∥}^{2}}$$
(B. 2)

where$\text{d}\in {\mathbb{R}}^{N\times 1}$is the spatial derivative image w.r.t. that motion axis.

## Appendix C The approximation to r(Δm,GS)

To interpretr(Δm,GS), we derived a mathematical approximation that reveals the key factors. We begin with the expression

$$r\left({\text{g}}_{s},\;\Delta \text{m}\right)=\frac{{\text{g}}_{s}^{T}\Delta \text{m}}{∥\Delta \text{m}∥\;∥{\text{g}}_{s}∥}=\frac{{\text{g}}_{s}^{T}\left({\text{m}}_{e2}-{\text{m}}_{e1}\right)}{∥\Delta \text{m}∥\;∥{\text{g}}_{s}∥}$$
(C. 1)

where${\text{g}}_{s}\in {\mathbb{R}}^{K\times 1}$,$\Delta \text{m}={\text{m}}_{e2}-{\text{m}}_{e1}\in {\mathbb{R}}^{K\times 1}$represent the GS and the difference between the e2 and e1 motion estimates, respectively, and${\text{m}}_{ei}\in {\mathbb{R}}^{K\times 1}$represents the motion estimates from theith echo. Then, using the approximation described inEq. B.2, we can write

$$\begin{array}{l} r(\Delta \text{m},\;GS)=\frac{{\text{g}}_{S}^{T}({\text{m}}_{e2}-{\text{m}}_{e1})}{\left||\Delta \text{m}||\;||{\text{g}}_{s}|\right|}\\ \;\;\;\;\;\;\;\;\;\;\;\;\;\;\;\;\;\;\;\;\;\;\;\;\;\;\;\approx \frac{{\text{g}}_{S}^{T}\left(\frac{{\text{Y}}_{e2}^{T}{\text{d}}_{e2}}{||{\text{d}}_{e2}|{|}^{2}}-\frac{{\text{Y}}_{e1}^{T}{\text{d}}_{e1}}{||{\text{d}}_{e1}|{|}^{2}}\right)}{\left||\Delta \text{m}||\;||{\text{g}}_{s}|\right|}\\ \;\;\;\;\;\;\;\;\;\;\;\;\;\;\;\;\;\;\;\;\;\;\;\;\;\;\;=\frac{\left||{\text{g}}_{s}|\right|}{\left||\Delta \text{m}|\right|}\left(\frac{{\text{g}}_{s}^{T}{\text{Y}}_{e2}^{T}{\text{d}}_{e2}}{||{\text{g}}_{s}|{|}^{2}||{\text{d}}_{e2}|{|}^{2}}-\frac{{\text{g}}_{s}^{T}{\text{Y}}_{e1}^{T}{\text{d}}_{e1}}{||{\text{g}}_{s}|{|}^{2}||{\text{d}}_{e1}|{|}^{2}}\right)\\ \;\;\;\;\;\;\;\;\;\;\;\;\;\;\;\;\;\;\;\;\;\;\;\;\;\;\;=\frac{\left||{\text{g}}_{s}|\right|}{\left||\Delta \text{m}|\right|}\left(\frac{\beta_{gs,\;e2}^{T}{\text{d}}_{e2}}{||{\text{d}}_{e2}|{|}^{2}}-\frac{\beta_{gs,\;e1}^{T}{\text{d}}_{e1}}{||{\text{d}}_{e1}|{|}^{2}}\right) \end{array}$$
(C. 2)

where$\beta_{{\text{g}}_{s}}{,}_{ei}=\frac{{\text{Y}}_{ei}^{T}{\text{g}}_{s}}{∥{\text{g}}_{s}{∥}^{2}}$is the GS beta coefficient map calculated from the linear fit of the GS to the unregistered functional data of theith echo.
