# Supplementary material for: Resting-state global brain activity induces bias in fMRI motion estimates
Source: Imaging Neurosci (Camb). 2024 May 6;2:imag-2-00151. doi: 10.1162/imag_a_00151 (PMC12247554; doi:10.1162/imag_a_00151)
Supplement: Supplementary Material [file imag_a_00151-supp.pdf]

Supplementary Material for "Resting state global brain activity induces bias in fMRI motion estimates"

Yixiang Mao[a,b,*], Conan Chen[a,b], Truong Nguyen[b], Thomas T. Liu[a,c,*]

[a] *Center for Functional MRI, University of California San Diego, 9500 Gilman Drive MC 0677, La Jolla, CA 92093*
[b] *Departments of Electrical and Computer Engineering, University of California San Diego, 9500 Gilman Drive, La Jolla, CA 92093*
[c] *Departments of Radiology, Psychiatry, and Bioengineering, University of California San Diego, 9500 Gilman Drive, La Jolla, CA 92093*

---

*Corresponding Authors
Email addresses: y1mao@ucsd.edu (Yixiang Mao), ttliu@ucsd.edu (Thomas T. Liu)

Preprint submitted to Imaging Neuroscience *April 1, 2024*

S1. Supplementary Methods

S1.1. Spatial correlation between the vigilance template and $\boldsymbol{\beta}_{\boldsymbol{g}_s}$

To investigate whether the asymmetric spatial patterns in $\boldsymbol{\beta}_{\boldsymbol{g}_s}$ are associated with vigilance-related rsfMRI signal fluctuations, we compared $\boldsymbol{\beta}_{\boldsymbol{g}_s}$ from the example run shown in Fig. 3 to the eyes-open vigilance template estimated in (Falahpour et al., 2018). Here, $\boldsymbol{\beta}_{\boldsymbol{g}_s}$ was calculated on the functional data after registration, transformation to MNI152 standard space, and spatial smoothing with a 4mm FWHM Gaussian kernel. The vigilance template in (Falahpour et al., 2018) was in the N27 template, so it was transferred to the MNI152 standard space in this work. The spatial correlation between the vigilance template and the example $\boldsymbol{\beta}_{\boldsymbol{g}_s}$ was calculated.

S1.2. Examining the intensity inhomogeneity in the fMRI data

Prior work has shown that the spatial intensity inhomogeneity (also known as the bias fields) in fMRI data can lead to registration errors (Gonzalez-Castillo et al., 2013). We found that the images in the dataset exhibited posterior-anterior intensity inhomogeneity, which may contribute to the posterior-anterior asymmetry pattern observed in $\boldsymbol{\beta}_{\boldsymbol{g}_s}$. To illustrate the effect of the intensity inhomogeneity, we picked two low motion and high aGS runs as examples, one with a high Ty $r(\Delta\boldsymbol{m}, \text{GS})$ value of 0.87 (the example run shown in Fig. S6) and another one with a low Ty $r(\Delta\boldsymbol{m}, \text{GS})$ value of 0.07 (this example run was not shown before). For each run, the spatial intensity inhomogeneity was estimated using the reference image (i.e., the first volume) with the segmentation function provided in SPM (Gispert et al., 2004).

S1.3. Comparing the GS-induced bias in motion estimates from AFNI, SPM, FSL and ANTS

In addition to AFNI *3dvolreg*, we also performed a preliminary examination of the $r(\Delta\boldsymbol{m}, \text{GS})$ values calculated with the motion estimates from three other commonly used algorithms: SPM *spm_realign* (Friston et al., 1995), FSL *mcflirt* (Jenkinson and Smith, 2001; Jenkinson et al., 2002), and ANTS *antsMotionCorr* (Avants et al., 2011, 2014).

For SPM *spm_realign* and FSL *mcflirt*, we generally used the default settings while making a few necessary modifications. For SPM, we disabled the default spatial smoothing of 5 mm FWHM by setting the parameter *fwhm* to 0, since we found that applying spatial smoothing before registration can increase the level of the GS-induced bias in the motion estimates. Also, we disabled registration to the mean

volume by setting the parameter *rtm* to 0. For FSL, we changed the reference volume to the first volume to be consistent with AFNI *3dvolreg*. As FSL supports different cost functions, in addition to the default cost function (normalized correlation, NC), we also estimated the motion parameters using the least-squares (LS) and mutual information (MI) cost functions. The LS cost function is used by AFNI and SPM, and the MI cost function has demonstrated the ability to reduce the neural-related bias in motion estimates (Freire and Mangin, 2001; Freire et al., 2002).

For ANTS *antsMotionCorr*, there is no default setting. Therefore, we followed the settings in an example that uses a rigid transformation and the MI cost function (github link). The first image was used as the reference image. It is worth mentioning that the sampling percentage was set to 10%, meaning that the algorithm only samples and uses 10% of the voxels during motion estimation. To make a fair comparison with the other algorithms, we also examined the motion parameters estimated using all the voxels (i.e., setting the sampling percentage to 100%).

For each run, the motion was estimated using both the e1 and e2 data. Because different algorithms have different default orientations, they output motion parameters in different orientations. To account for these differences, we multiplied the motion parameters by either 1 or -1 so that the resulting motion parameters were in the RAI orientation. The mean and the linear and quadratic trends were regressed out from the motion estimates.

Following the descriptions in Methods 2.4, for each run and motion axis, $r(\Delta \boldsymbol{m}, \mathrm{GS})$ was calculated and the significance of the $r(\Delta \boldsymbol{m}, \mathrm{GS})$ values was assessed by empirical null distributions. The mean $r(\Delta \boldsymbol{m}, \mathrm{GS})$ values over runs and the percent of runs showing significant $r(\Delta \boldsymbol{m}, \mathrm{GS})$ values were calculated to assess the level of the GS-induced bias in the motion estimates.

S2. Supplementary Results

S2.1. $r(\Delta \boldsymbol{m}, GS)$ calculated using motion estimates with AFNI default weightings and GS after registration

In the main text, when we calculated $r(\Delta \boldsymbol{m}, \mathrm{GS})$, we used the motion parameters estimated without AFNI default weightings and the GS calculated before volume registration. Here, we examined how these choices affect the $r(\Delta \boldsymbol{m}, \mathrm{GS})$ values. Fig. S2 shows comparisons of (a) mean $r(\Delta \boldsymbol{m}, \mathrm{GS})$ over runs and (b) the percentage of runs showing significant $r(\Delta \boldsymbol{m}, \mathrm{GS})$ values calculated with the motion parameters estimated with and without weightings (denoted as $\mathrm{Mot}_{\mathrm{wt}}$ and Mot, respectively) and the GS calculated

before and after volume registration (denoted as GS and GS_{vr}, respectively). Results obtained with GS and GS_{vr} exhibit similar values for both the mean $r(\Delta\boldsymbol{m}, \text{GS})$ and the percentage of runs showing significant $r(\Delta\boldsymbol{m}, \text{GS})$. Comparing motion estimates with and without default weightings, we observe that Mot_{wt} shows higher percentages of runs showing significant $r(\Delta\boldsymbol{m}, \text{GS})$ values in all motion axes except for the Ty axis. In the Ty axis, Mot_{wt} shows a slight decrease (about 1%) in the percentage of runs showing significant $r(\Delta\boldsymbol{m}, \text{GS})$ values. These results suggest that using AFNI default weightings may lead to a slightly higher level of the bias in the motion estimates in all motion axes except for the Ty axis.

S2.2. Effect of the bias on ROI-ROI FC via motion regression without motion censoring

The differences in the ROI-ROI FC calculated after e2 and e1 motion regression (i.e., Δz) without motion censoring are plotted in Fig. S7 and compared to Δz calculated with motion censoring in Fig. S8. In Fig. S7, we observe that the range and pattern of the average Δz values obtained without censoring are similar to those of the average Δz values obtained with censoring shown in Fig. 5.

Fig. S8 shows two-sided violin plots of the distributions of mean Δz (e2-e1) over ROI pairs calculated with (blue) and without (green) motion censoring for the four groups of runs. For each group, a paired t-test was calculated to compare the mean Δz with and without motion censoring. However, no significant ($p > 0.01$) differences were found in any of the four groups.

Taken together, these results suggest that motion censoring has little impact on the changes in rsFC estimates due to regression with biased motion estimates.

S2.3. Spatial correlation between the vigilance template and $\boldsymbol{\beta}_{\boldsymbol{g}_s}$

Fig. S20 compares $\boldsymbol{\beta}_{\boldsymbol{g}_s}$ from the example run shown in Fig. 3 to the eyes-open vigilance template estimated in (Falahpour et al., 2018) (both spatial maps were transferred to the MNI152 standard space). As shown in Fig. S20 (b), the estimated vigilance template exhibits similar superior-inferior and posterior-anterior asymmetric spatial patterns to the example $\boldsymbol{\beta}_{\boldsymbol{g}_s}$ map, resulting in a strong negative correlation ($r = -0.38$, $p < 1 \times 10^{-6}$) between $\boldsymbol{\beta}_{\boldsymbol{g}_s}$ and the vigilance template. The observed negative correlation is consistent with the negative correlations between the GS and the EEG vigilance found in (Falahpour et al., 2016, 2018). Hence, the asymmetric spatial patterns in $\boldsymbol{\beta}_{\boldsymbol{g}_s}$ may be linked to resting-state global brain activity associated with vigilance changes.

S2.4. Examining the intensity inhomogeneity in the fMRI data

Fig. S21 shows the reference images and estimated bias field maps from the example runs. For the example run with a strong $r(\Delta\boldsymbol{m}, \mathrm{GS})$ in the Ty axis shown in Fig. S21 (a), its reference image and estimated bias field map exhibit higher values at the posterior part of the brain and lower values at the anterior part of the brain. In contrast, for the run with a Ty $r(\Delta\boldsymbol{m}, \mathrm{GS})$ value close to zero (shown in Fig. S21 (b)), its reference image and estimated bias field map show comparable values at the anterior and posterior parts of the brain. These results suggest that the intensity inhomogeneity in the fMRI data used for this study may contribute to the posterior-anterior asymmetric pattern in $\boldsymbol{\beta}_{\boldsymbol{g}_s}$. In addition, although both runs showed high positive $r(\Delta\boldsymbol{m}, \mathrm{GS})$ values in the Tz axis, their estimated bias field maps showed minimal superior-inferior asymmetry, suggesting that the intensity inhomogeneity is not contributing to the superior-inferior asymmetry in $\boldsymbol{\beta}_{\boldsymbol{g}_s}$. Because spatial intensity inhomogeneity in fMRI data is affected by multiple factors, including radio-frequency coil homogeneity, the pulse sequences used, and the imaged object itself (Belaroussi et al., 2006), the impact of the intensity inhomogeneity on motion bias may vary greatly across datasets.

S2.5. Assess the GS-induced bias in motion estimates from SPM, FSL and ANTS

Fig. S22 (a) and (b) show the mean $r(\Delta\boldsymbol{m}, \mathrm{GS})$ values and the percent of runs showing significant $r(\Delta\boldsymbol{m}, \mathrm{GS})$ values, respectively. For all algorithms and cost functions, the Ty and Tz axes exhibit higher mean $r(\Delta\boldsymbol{m}, \mathrm{GS})$ values and a higher percentage of runs showing significant $r(\Delta\boldsymbol{m}, \mathrm{GS})$ values as compared to the other motion axes, consistent with the results in the main text. As the mean $r(\Delta\boldsymbol{m}, \mathrm{GS})$ values and the percent of runs showing significant $r(\Delta\boldsymbol{m}, \mathrm{GS})$ values follow a similar pattern, we will focus on the mean $r(\Delta\boldsymbol{m}, \mathrm{GS})$ values.

In the Tz axis, when comparing different cost functions, the LS cost function shows higher mean $r(\Delta\boldsymbol{m}, \mathrm{GS})$ values as compared to the NC and MI cost functions. When comparing across algorithms, AFNI (LS) and SPM (LS) show comparably high mean $r(\Delta\boldsymbol{m}, \mathrm{GS})$ values, FSL with different cost functions (LS, NC and MI) show moderate mean $r(\Delta\boldsymbol{m}, \mathrm{GS})$ values, and ANTS (MI) shows the lowest mean $r(\Delta\boldsymbol{m}, \mathrm{GS})$ values. It is worth noting that for ANTS (MI), using 10% of the voxels during registration shows a lower mean $r(\Delta\boldsymbol{m}, \mathrm{GS})$ value as compared to using all the voxels. In the Ty axis, AFNI (LS) shows the highest mean $r(\Delta\boldsymbol{m}, \mathrm{GS})$ value, SPM (LS) and FSL with different cost functions show moderate mean $r(\Delta\boldsymbol{m}, \mathrm{GS})$ values, and ANTS (MI) shows the lowest mean $r(\Delta\boldsymbol{m}, \mathrm{GS})$ values. Overall, we

observe that both the software package and cost function affect the level of the bias, with differences in the software implementation of the same cost function playing a significant role.

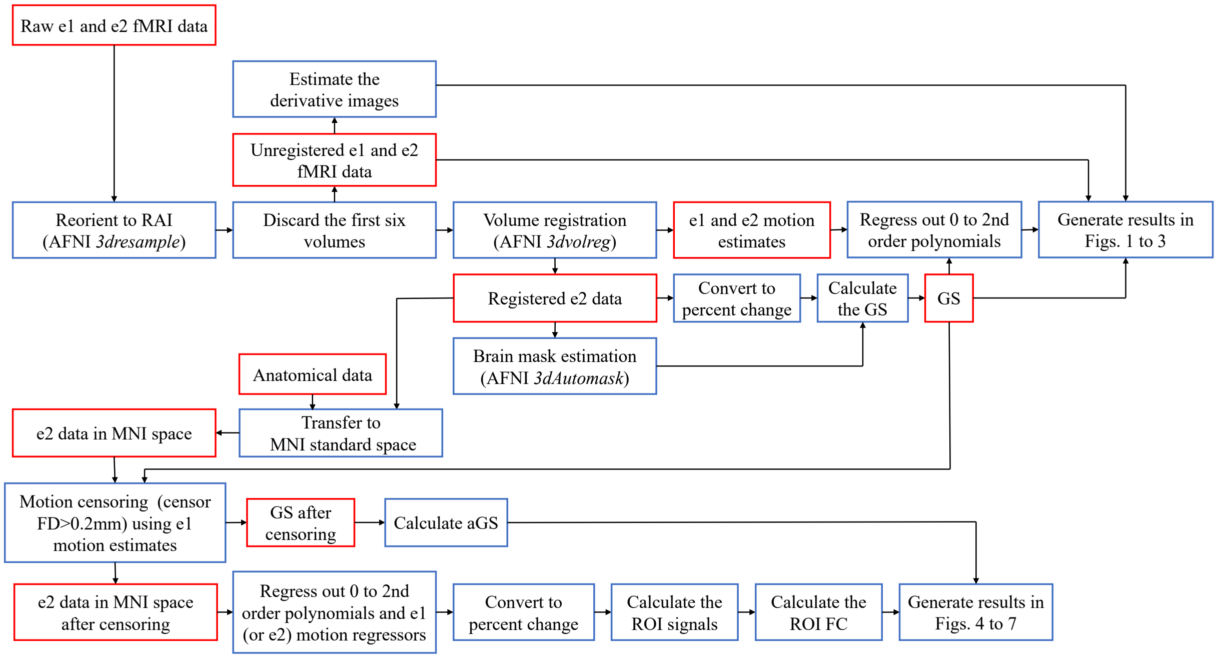

Figure S1: Diagram of processing workflow. See text for detailed descriptions.

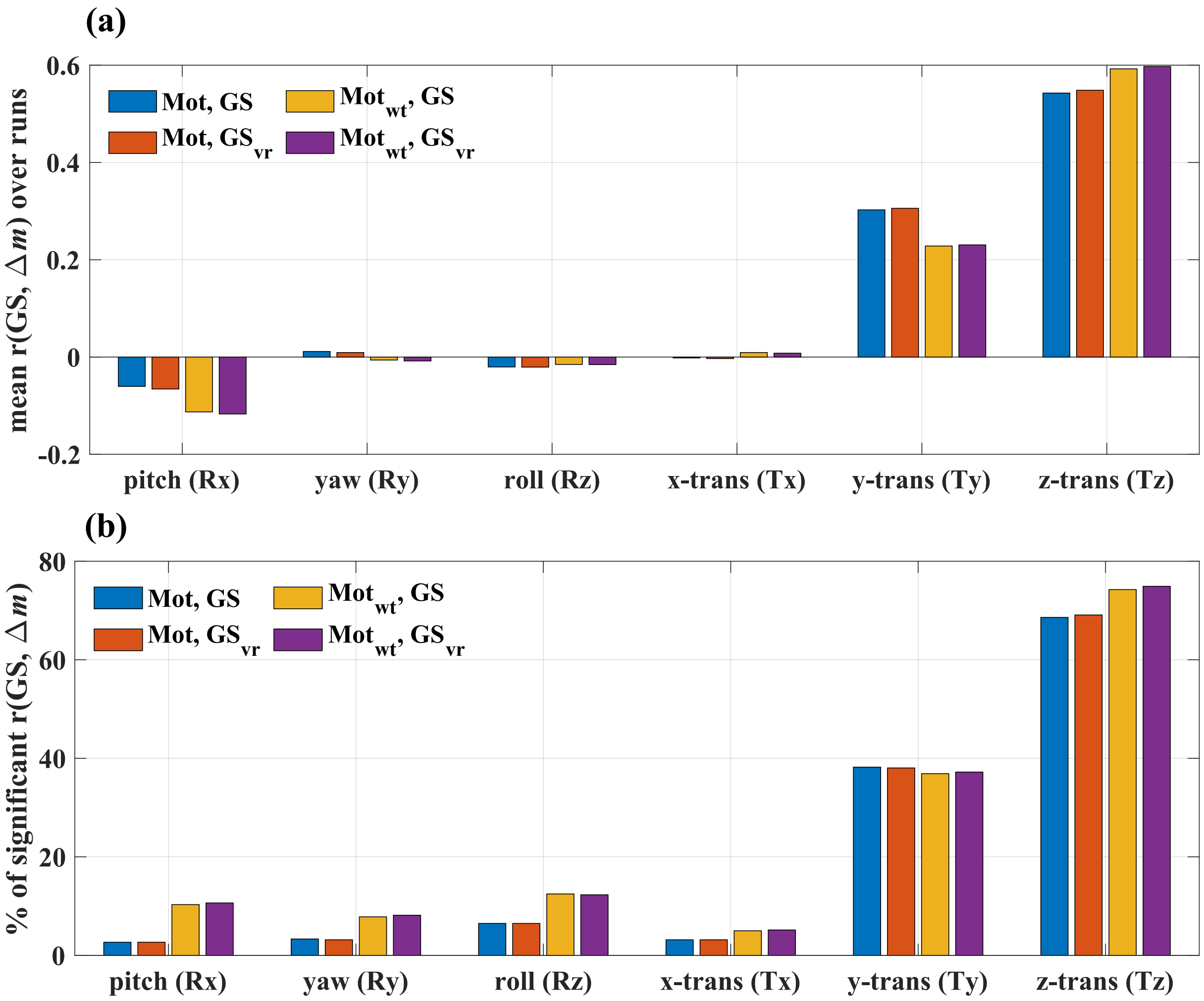

Figure S2: Comparisons of (a) mean $r(\Delta\boldsymbol{m}, \mathrm{GS})$ over runs and (b) the percentage of runs showing significant $r(\Delta\boldsymbol{m}, \mathrm{GS})$ values calculated with combinations of the motion parameters estimated with and without weightings (denoted as Mot and $\mathrm{Mot}_{\mathrm{wt}}$, respectively) and the GS calculated before and after volume registration (denoted as GS and $\mathrm{GS}_{\mathrm{vr}}$, respectively). The $r(\Delta\boldsymbol{m}, \mathrm{GS})$ values were calculated after e1 motion regression. For each motion axis and combination of the GS and motion estimates, an empirical null distribution of $r(\Delta\boldsymbol{m}, \mathrm{GS})$ was generated by the permutation-based method and used to assess the significance of the measured $r(\Delta\boldsymbol{m}, \mathrm{GS})$ values. A Bonferroni-corrected run-wise p-value threshold of 0.05/602 was used, where 602 is the number of runs.

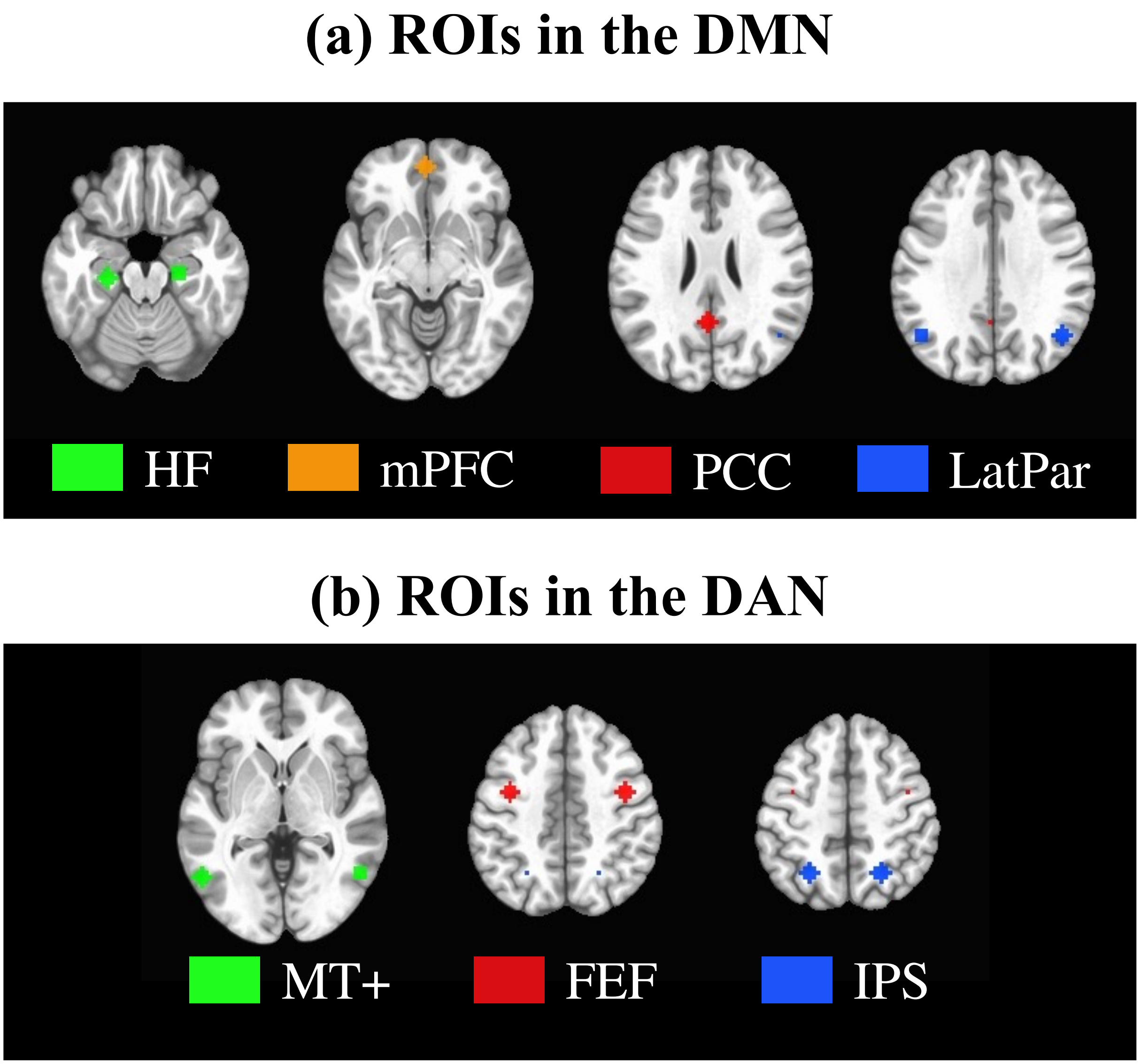

Figure S3: Depiction of four ROIS in the Default mode network (DMN): posterior cingulate cortex (PCC), lateral parietal cortex (LatPar), medial prefrontal cortex (mPFC) and Hippocampal formation (HF); and three ROIs in the dorsal attention network (DAN): frontal eye field (FEF), intraparietal cortex (IPS) and middle temporal area (MT+).

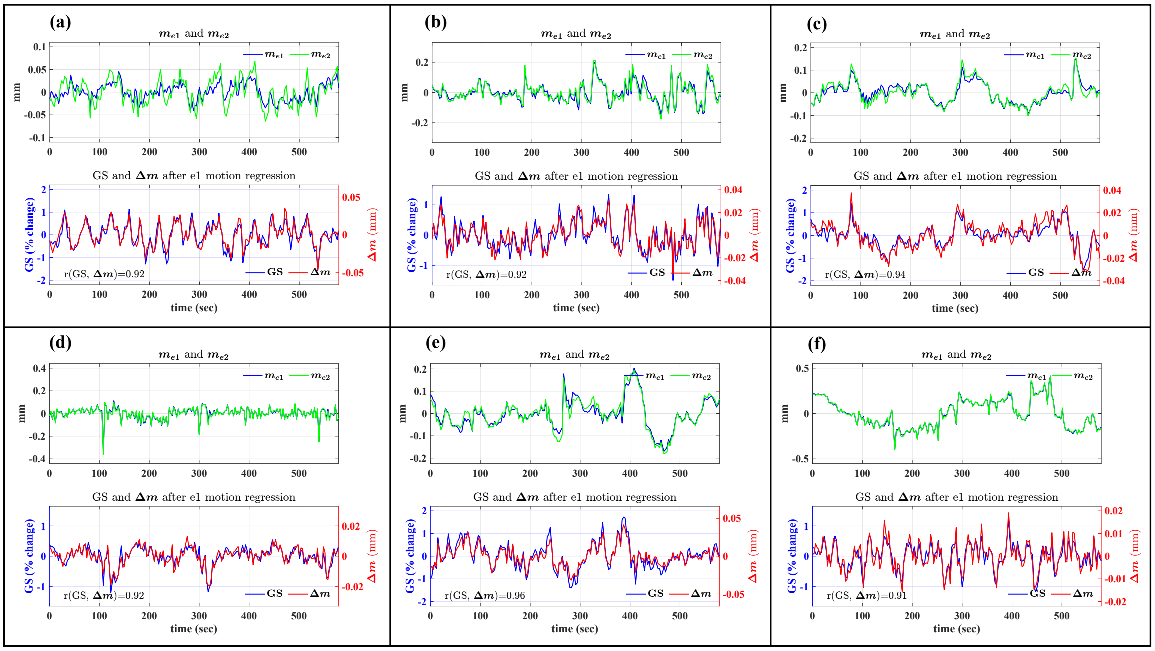

Figure S4: Global signal (GS) and motion estimates in the Tz axis, including $\boldsymbol{m}_{e1}$, $\boldsymbol{m}_{e2}$ and $\Delta\boldsymbol{m}$ from six example runs showing high $r(\Delta\boldsymbol{m}, \text{GS})$. The e1 motion regressors were regressed out from the GS and $\Delta\boldsymbol{m}$.

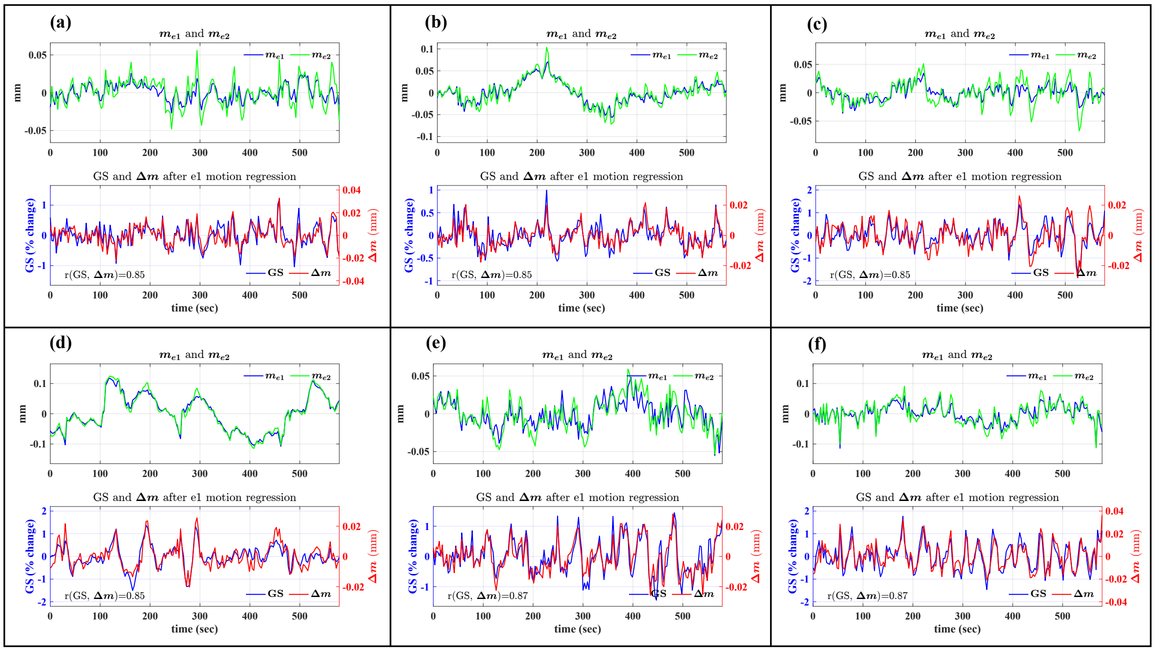

Figure S5: Global signal (GS) and motion estimates in the Ty axis, including $\boldsymbol{m}_{e1}$, $\boldsymbol{m}_{e2}$ and $\Delta\boldsymbol{m}$ from six example runs showing high $r(\Delta\boldsymbol{m}, \mathrm{GS})$. The e1 motion regressors were regressed out from the GS and $\Delta\boldsymbol{m}$.

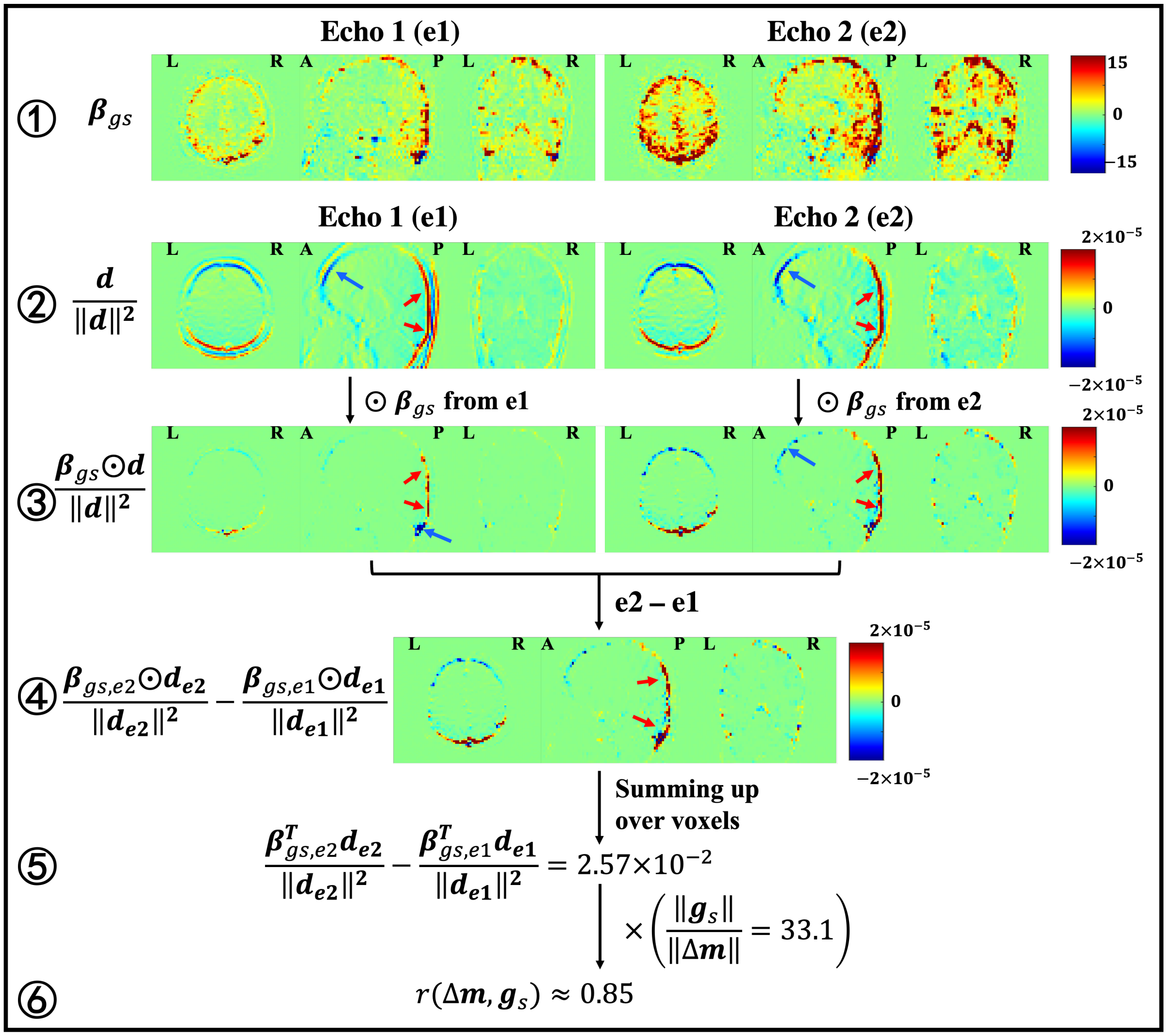

Figure S6: Visualization of the spatial maps underlying $r(\Delta \boldsymbol{m}, \text{GS})$ in the Ty axes from an example run. For each map, three representative slices (one axial, one sagittal and one coronal) are plotted. From top to bottom, rows 1 through 3 show the $\boldsymbol{\beta}_{\boldsymbol{g}_s}$, $\frac{\boldsymbol{d}}{\|\boldsymbol{d}\|^2}$, and $\frac{\boldsymbol{\beta}_{\boldsymbol{g}_s} \odot \boldsymbol{d}}{\|\boldsymbol{d}\|^2}$ maps from e1 and e2, where $\odot$ represents element-wise multiplication. Row 4 shows $\frac{\boldsymbol{\beta}_{\boldsymbol{g}_s} \odot \boldsymbol{d}}{\|\boldsymbol{d}\|^2}$ difference (e2-e1) maps. The red and blue arrows point to the brain regions showing high positive and negative values in the maps, respectively.

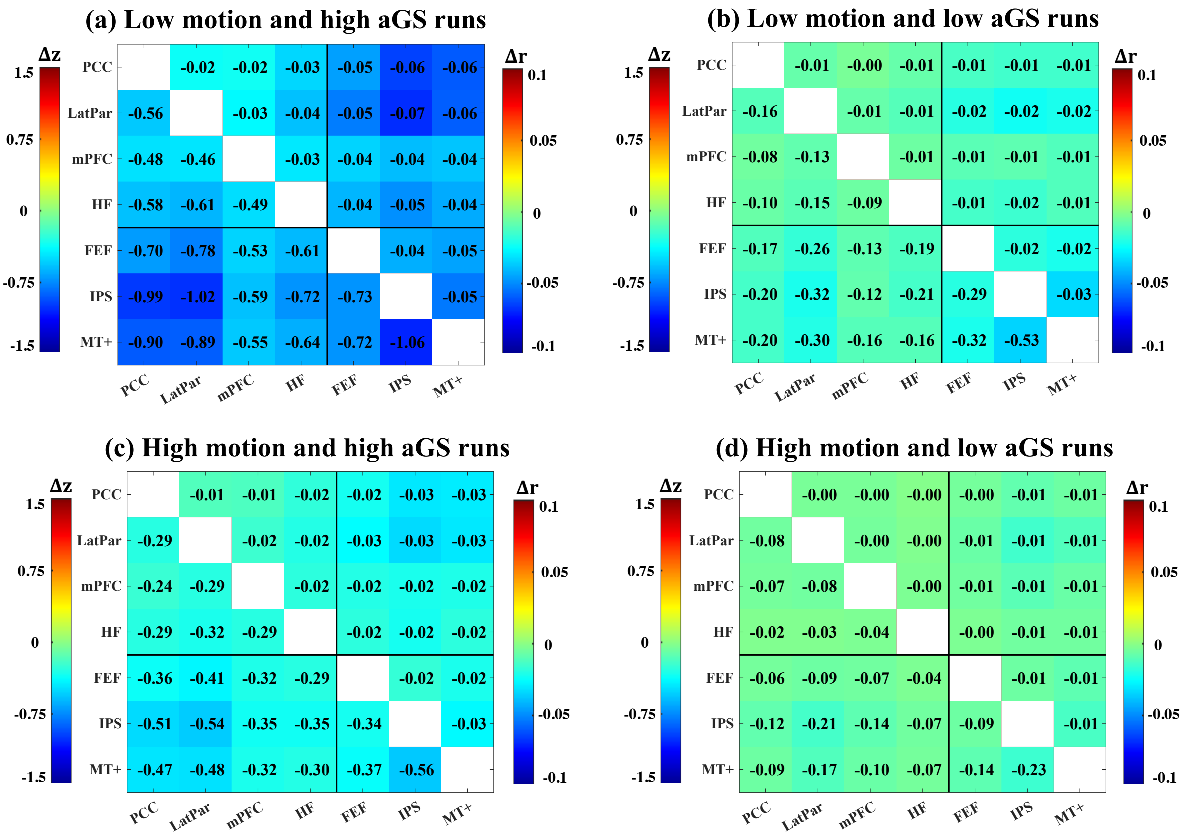

Figure S7: Differences (e2-e1) in ROI-ROI FC calculated after regressing out e1 and e2 motion regressors without motion censoring. The differences were averaged over runs in each of four groups: (a) low motion and high aGS runs, (b) low motion and low aGS runs, (c) high motion and high aGS runs and (d) high motion and low aGS runs. Each subplot is divided into an upper right triangle showing the average differences in r-values and a lower left triangle showing the average differences in z-scores.

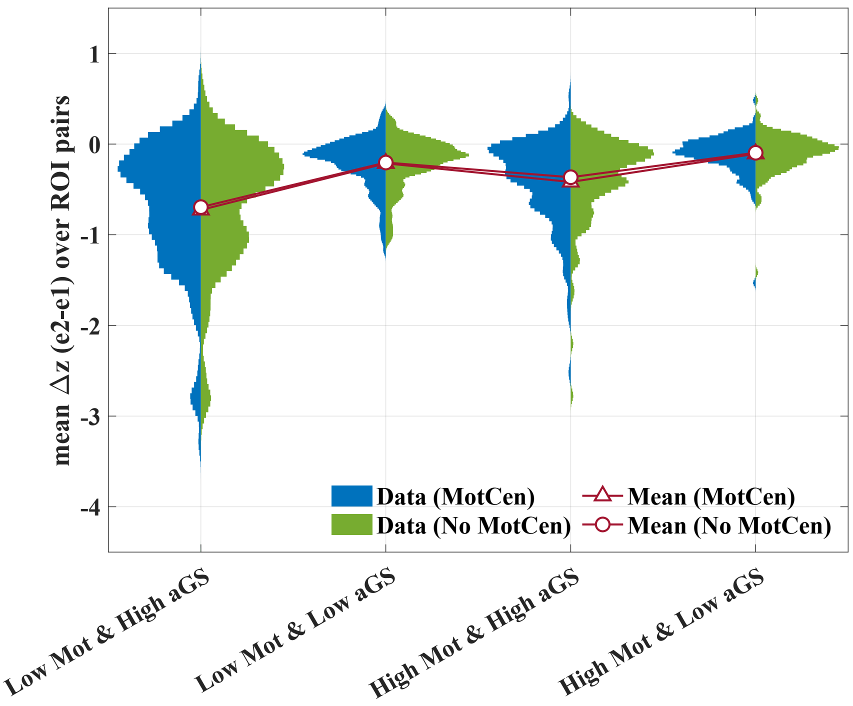

Figure S8: Two-sided violin plots showing the distributions of mean Δz (e2-e1) over ROI pairs calculated with (blue) and without (green) motion censoring for (1) low motion and high aGS runs, (2) low motion and low aGS runs, (3) high motion and high aGS runs, and (4) high motion and low aGS runs, where Δz indicates the differences (e2-e1) in ROI-ROI FC calculated after regressing out e1 and e2 motion regressors from the e2 data prior to computation of FC. The triangles and circles represent the mean of the distributions. For each group, a paired t-test was calculated to compare the mean Δz values with and without motion censoring. No significant difference was found in any of the four groups.

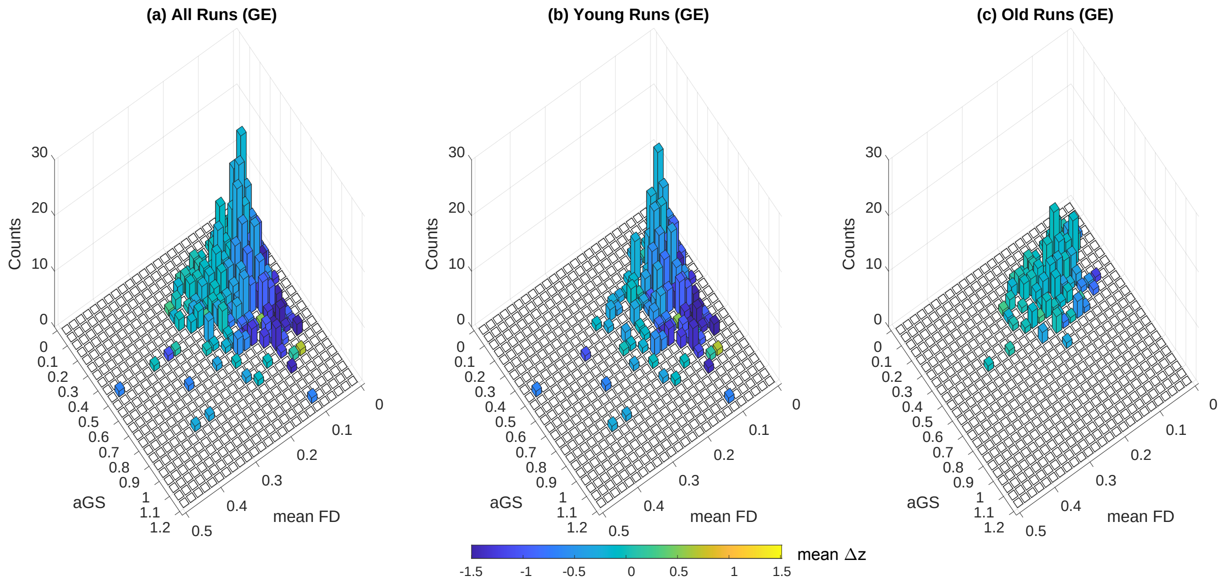

Figure S9: 2D scaled histograms (GE runs only) showing differences (e2-e1) in mean ROI-ROI FC (z-scores) as a function of aGS and mean FD (mm), where the heights of the bars indicates the count and the color indicates the mean Δz values.

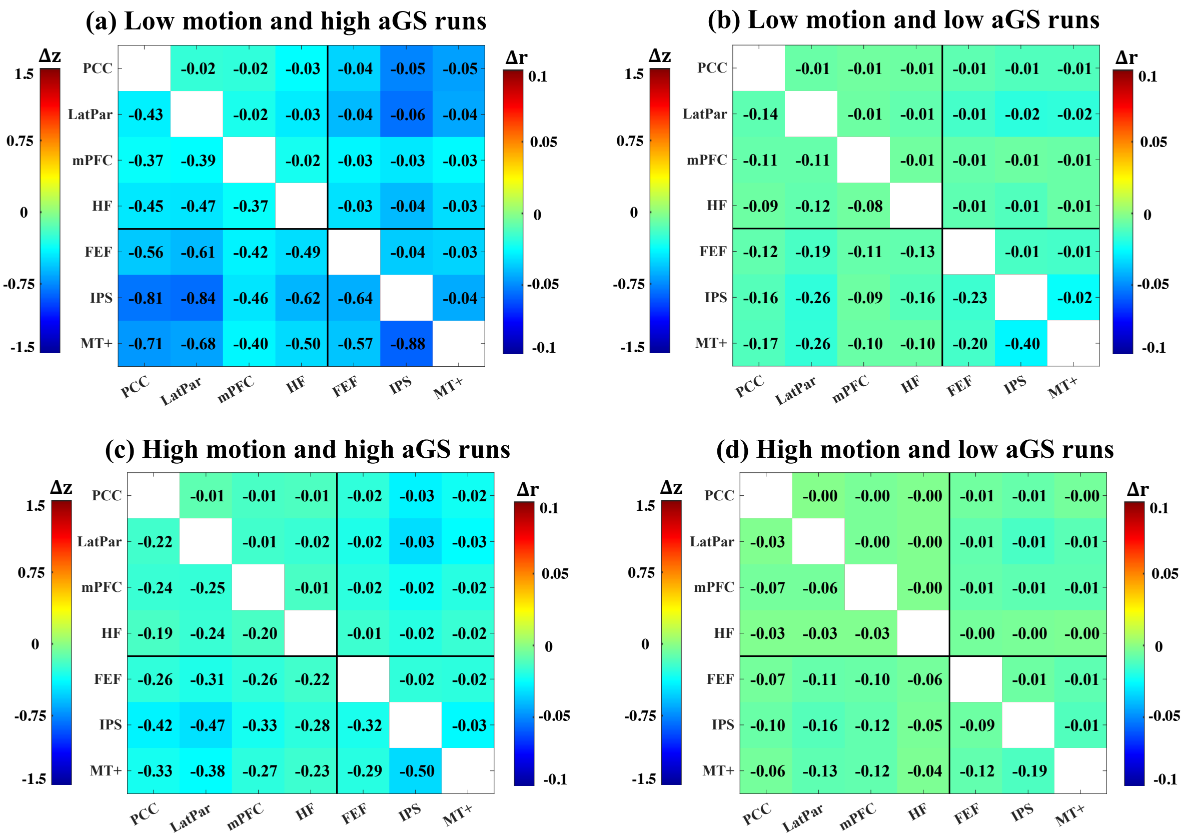

Figure S10: Differences (e2-e1) in ROI-ROI FC calculated after regressing out e1 and e2 motion regressors when including only Ty and Tz motion regressors. The differences were averaged over runs in each of four groups: (a) low motion and high aGS runs, (b) low motion and low aGS runs, (c) high motion and high aGS runs and (d) high motion and low aGS runs. Each subplot is divided into an upper right triangle showing the average differences in r-values and a lower left triangle showing the average differences in z-scores.

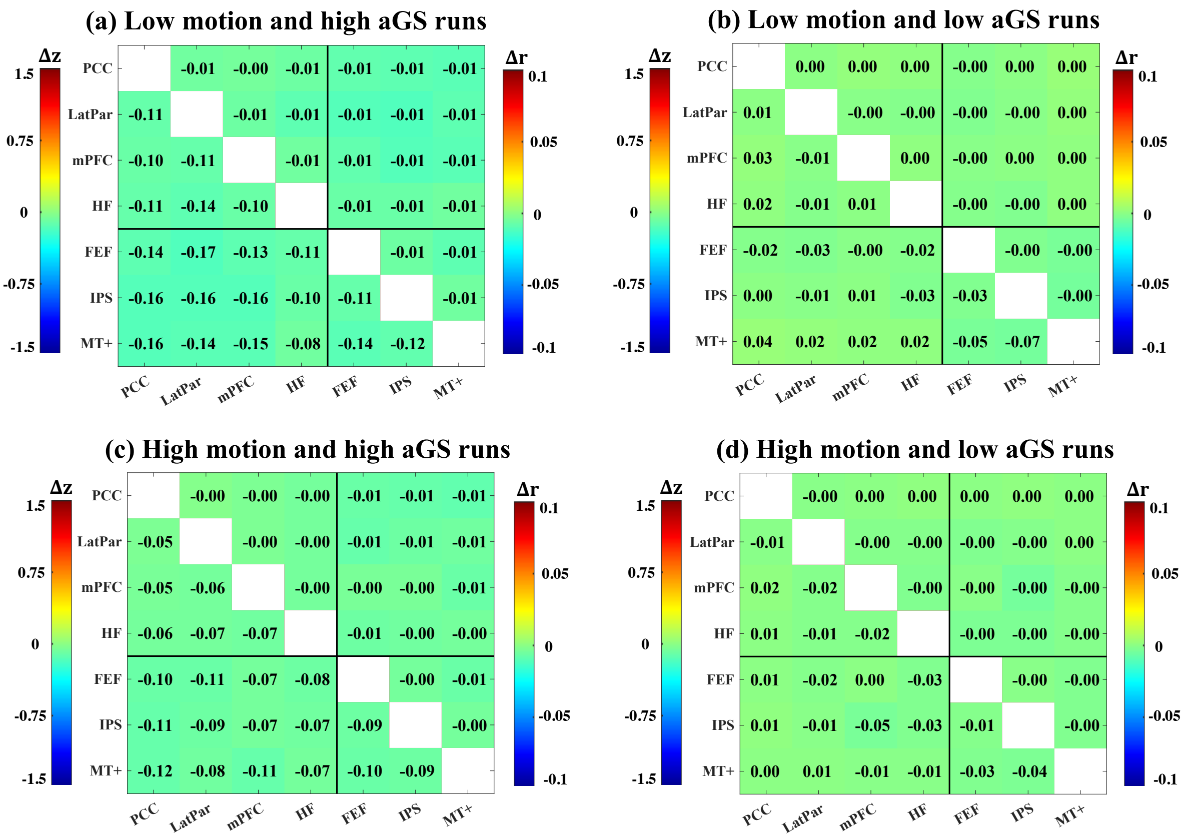

Figure S11: Differences (e2-e1) in ROI-ROI FC calculated after regressing out e1 and e2 motion regressors when excluding Ty and Tz motion regressors. The differences were averaged over runs in each of four groups: (a) low motion and high aGS runs, (b) low motion and low aGS runs, (c) high motion and high aGS runs and (d) high motion and low aGS runs. Each subplot is divided into an upper right triangle showing the average differences in r-values and a lower left triangle showing the averaged differences in z-scores.

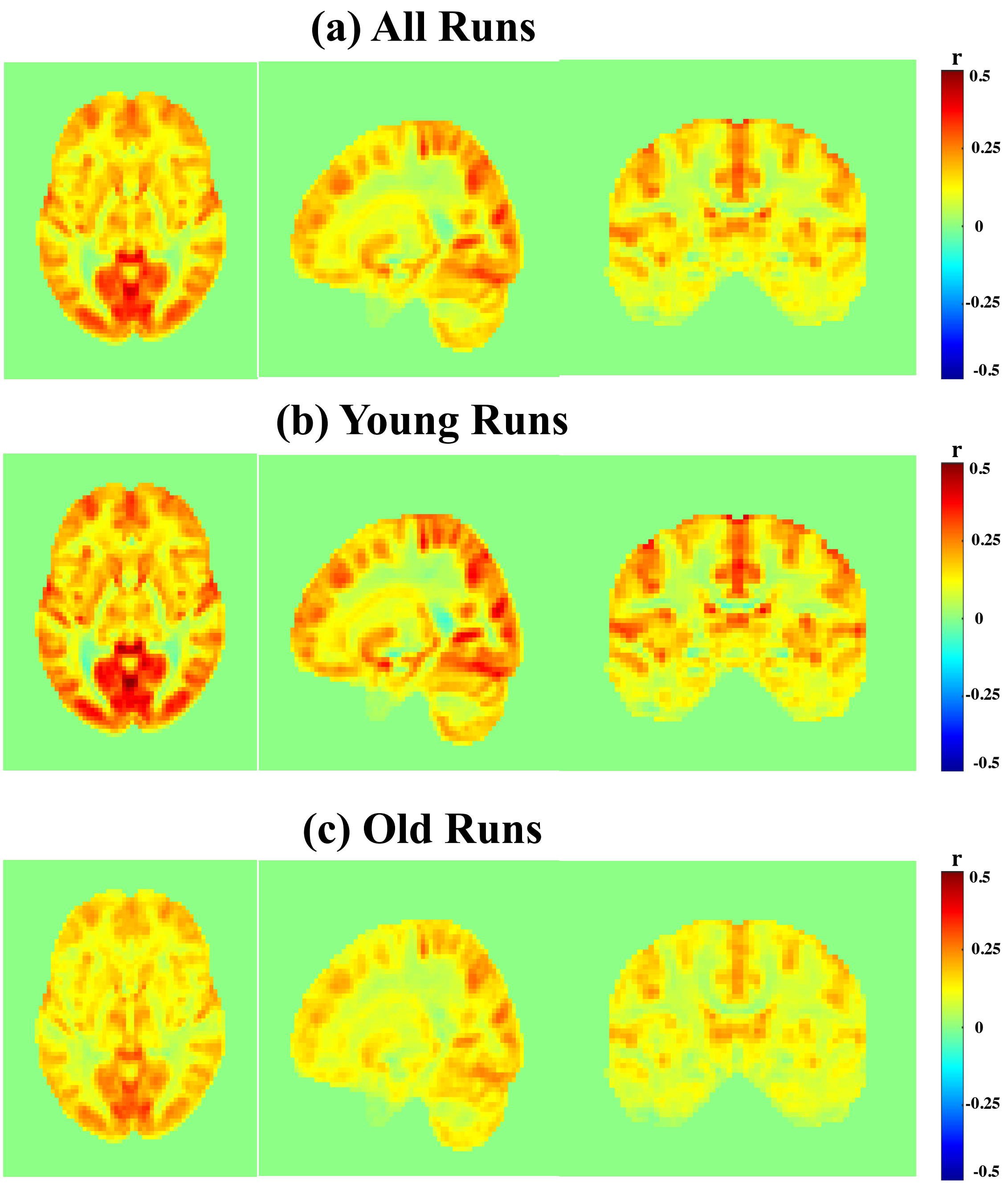

Figure S12: GS topography maps showing the correlations of the GS with each of the voxel time courses in the brain averaged over subjects in the indicated group.

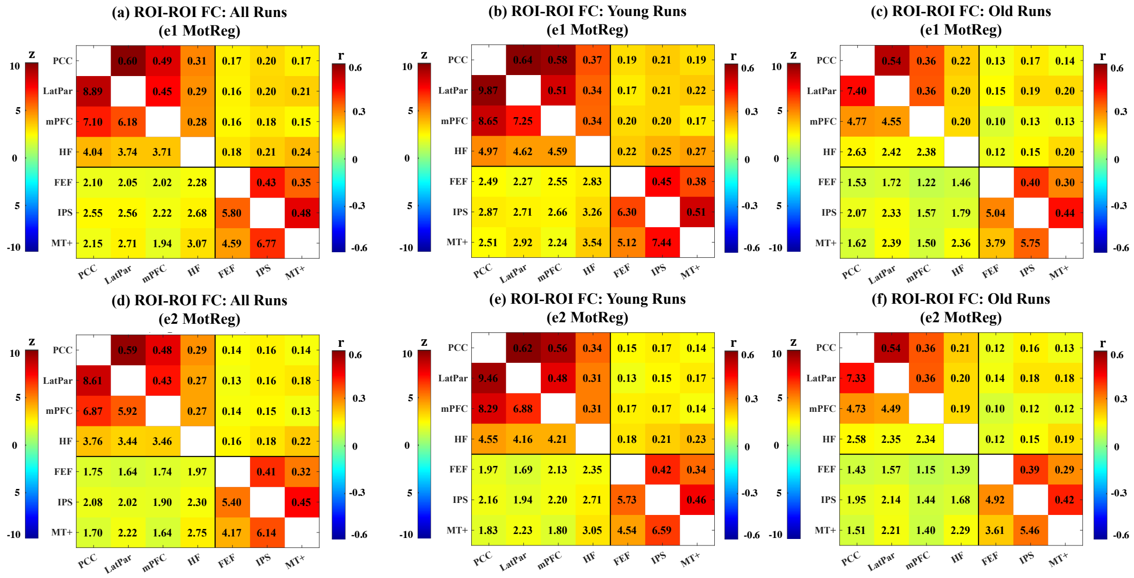

Figure S13: RSFC maps with e1 motion regression (top row) and e2 motion regression (bottom row).

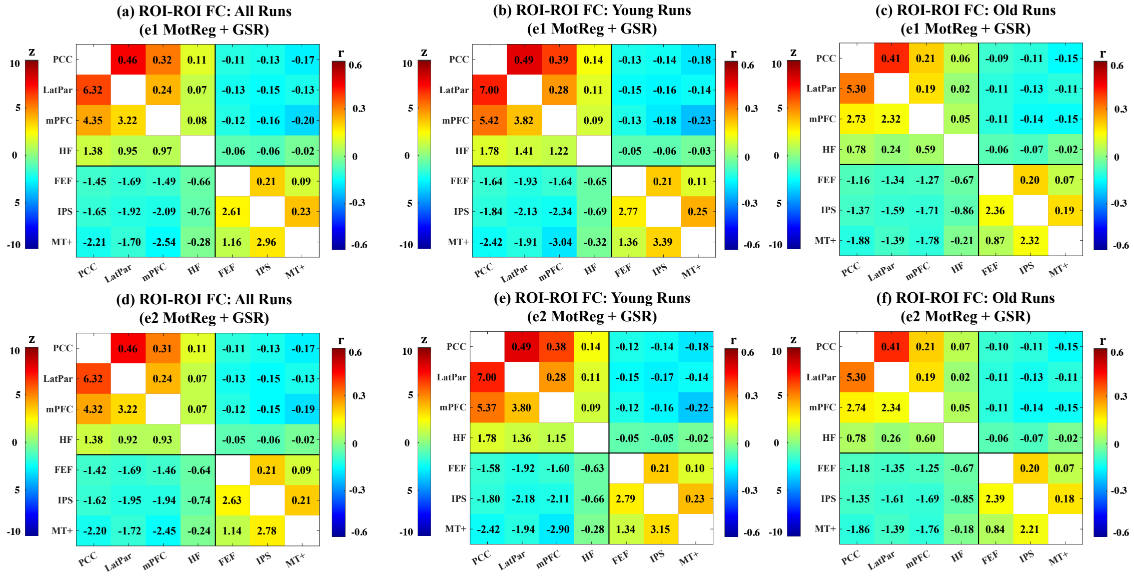

Figure S14: RSFC maps with e1 motion and global signal regression (top row) and e2 motion and global signal regression (bottom row).

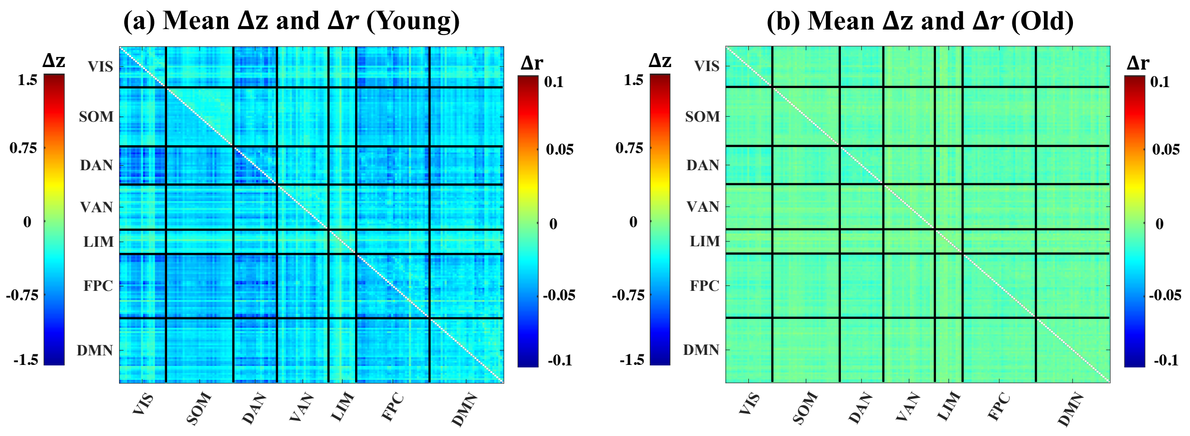

Figure S15: Mean Δz and Δr over (a) young and (b) old subjects for all ROI pairs using a 200-region parcellation of the brain. Regions are grouped into seven networks: VIS = visual, SOM = somatomotor, DAN = dorsal attention, VAN = ventral attention, LIM = limbic, FPC = frontoparietal control, DMN = default (Schaefer et al., 2018; Setton et al., 2022). Each subplot is divided into an upper right triangle showing the average differences in r-values and a lower left triangle showing the average differences in z-scores.

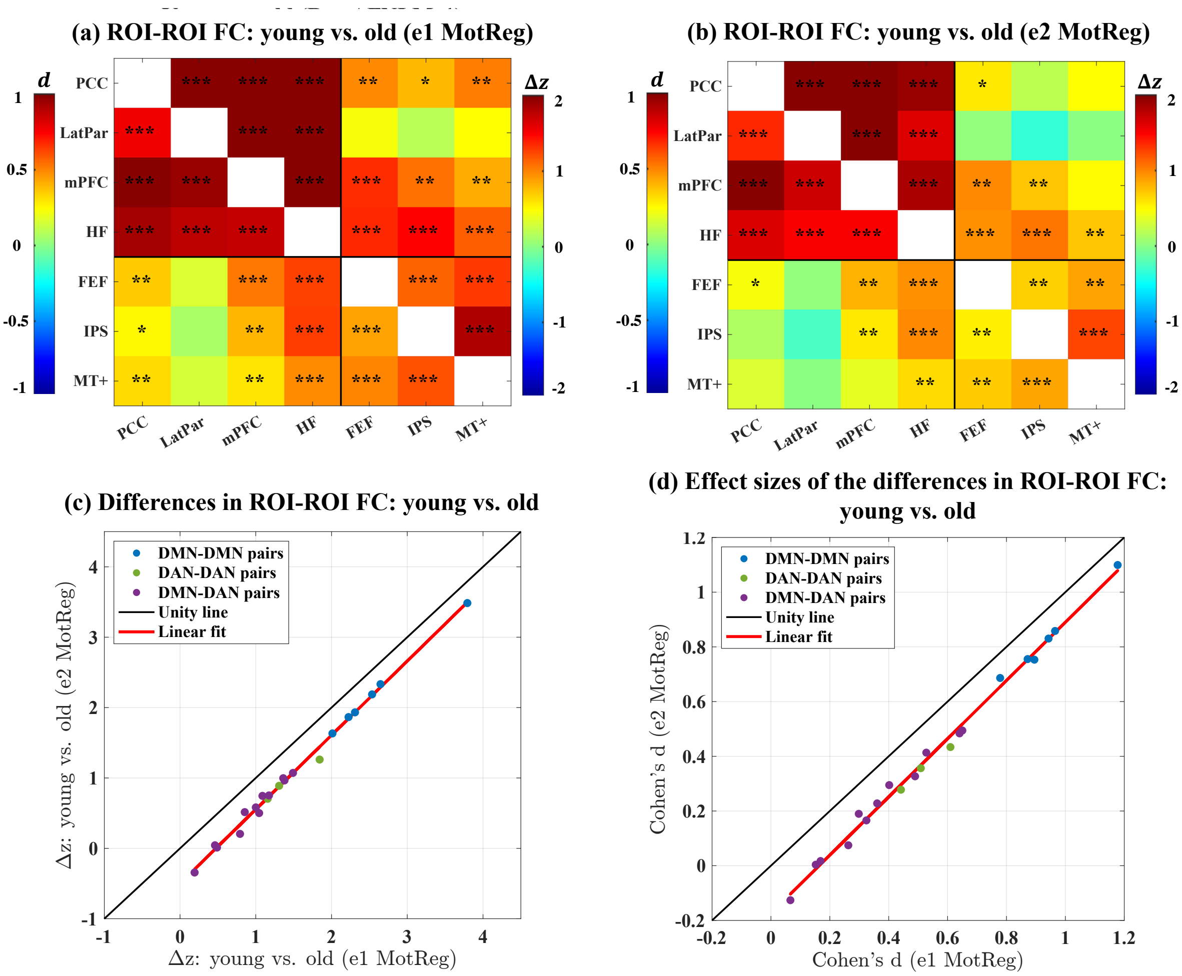

Figure S16: (a-b) ROI-ROI FC differences (young-old) between young and old subjects calculated after (a) e1 and (b) e2 motion regression, where transformation to standard space was performed using a custom young-old template (Laurita et al., 2020). Each subplot is divided into an upper right triangle showing the averaged differences in z-scores between young and old subjects and a lower left triangle showing the effect size of the differences. The black asterisks indicate the statistical significance of the differences assessed by permutation tests (*: $p < 0.01$, **: $p < 1 \times 10^{-3}$, ***: $p < 1 \times 10^{-6}$). A positive value (red color) indicates that the young subjects show higher connectivity as compared to the old subjects. (c) Scatter plot comparing the differences in ROI-ROI FC between the young and the old subjects calculated after e1 and e2 motion regression (denoted as e1 MotReg and e2 MotReg, respectively) for all pairs of ROIs. (d) Scatter plot comparing the effect sizes of the differences in ROI-ROI FC between the young and the old subjects calculated after e1 and e2 motion regression for all pairs of ROIs.

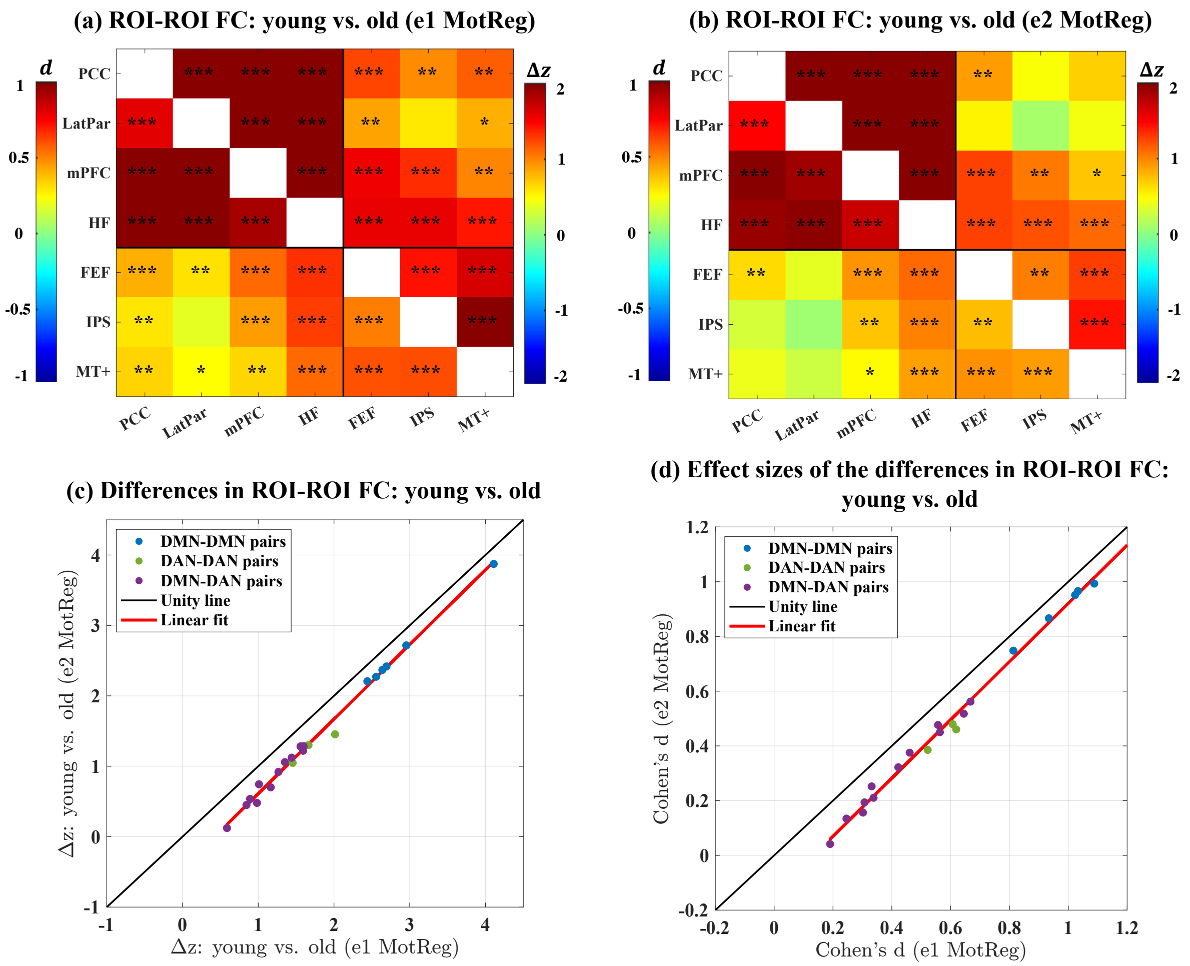

Figure S17: The effect of motion regression on the young vs. old FC differences (young-old) when including only the Ty and Tz motion estimates. (a-b) ROI-ROI FC differences between young and old subjects calculated after (a) e1 and (b) e2 motion regression. Each subplot is divided into an upper right triangle showing the average differences in z-scores between young and old subjects and a lower left triangle showing the effect size of the differences. The black asterisks indicate the statistical significance of the differences assessed by permutation tests (*: $p < 0.01$, **: $p < 1 \times 10^{-3}$, ***: $p < 1 \times 10^{-6}$). A positive value (red color) indicates that the young subjects show higher connectivity as compared to the old subjects. (c) Scatter plot comparing the differences in ROI-ROI FC between the young and the old subjects calculated after e1 and e2 motion regression for all pairs of ROIs. (d) Scatter plot comparing the effect size of the differences in ROI-ROI FC between the young and the old subjects calculated after e1 and e2 motion regression for all pairs of ROIs.

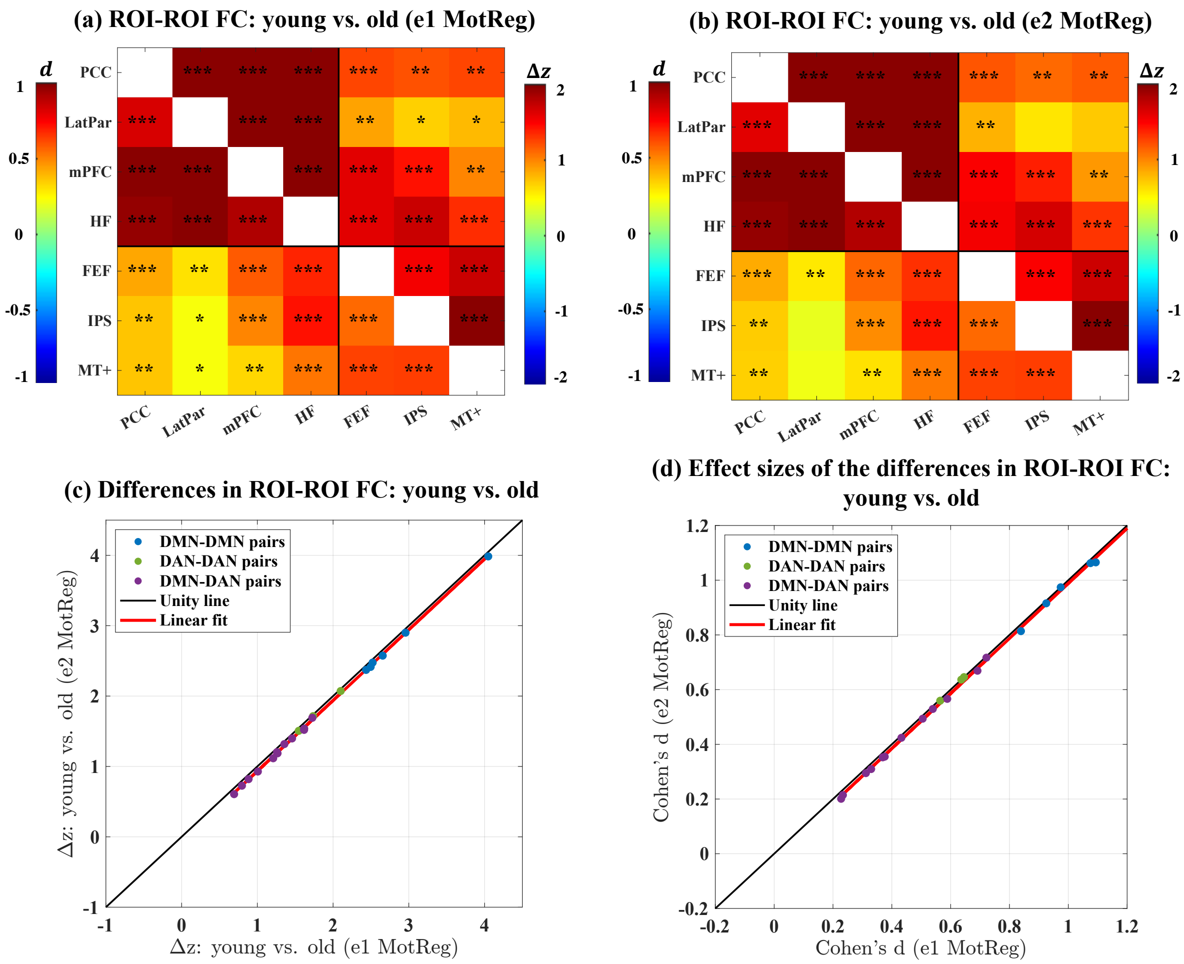

Figure S18: The effect of motion regression on the young vs. old FC differences (young-old) when excluding the Ty and Tz motion estimates. (a-b) ROI-ROI FC differences between young and old subjects calculated after (a) e1 and (b) e2 motion regression. Each subplot is divided into an upper right triangle showing the average differences in z-scores between young and old subjects and a lower left triangle showing the effect size of the differences. The black asterisks indicate the statistical significance of the differences assessed by permutation tests (*: $p < 0.01$, **: $p < 1 \times 10^{-3}$, ***: $p < 1 \times 10^{-6}$). A positive value (red color) indicates that the young subjects show higher connectivity as compared to the old subjects. (c) Scatter plot comparing the differences in ROI-ROI FC between the young and the old subjects calculated after e1 and e2 motion regression for all pairs of ROIs. (d) Scatter plot comparing the effect size of the differences in ROI-ROI FC between the young and the old subjects calculated after e1 and e2 motion regression for all pairs of ROIs.

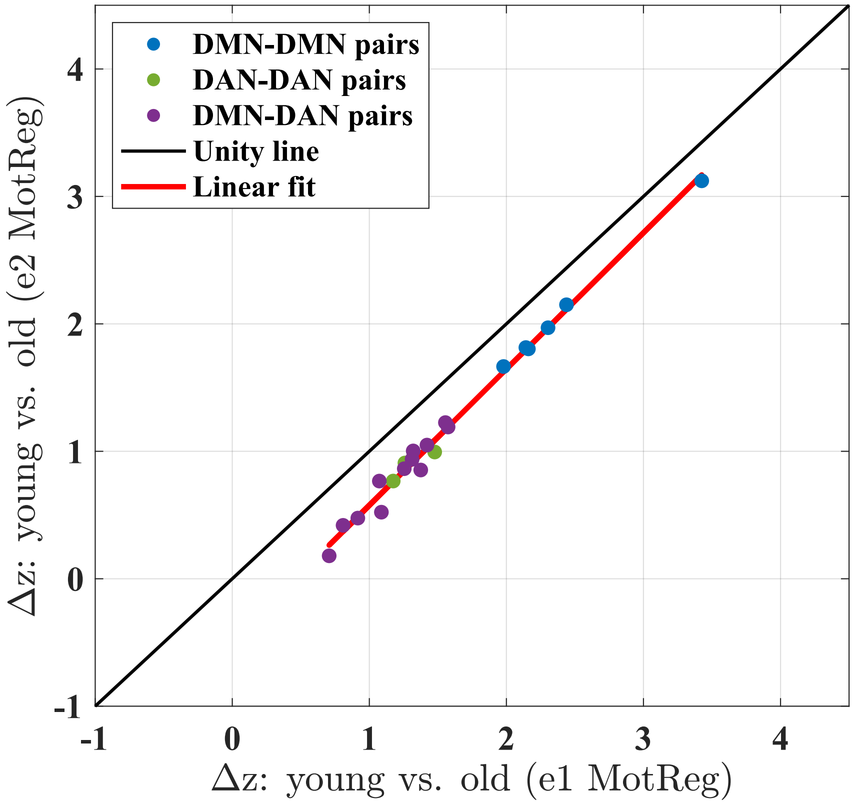

Figure S19: Differences in ROI-ROI FC between the young and the old subjects calculated after e1 and e2 motion regression (denoted as e1 MotReg and e2 MotReg, respectively) for all pairs of ROIs using an expanded model with FD as a confound.

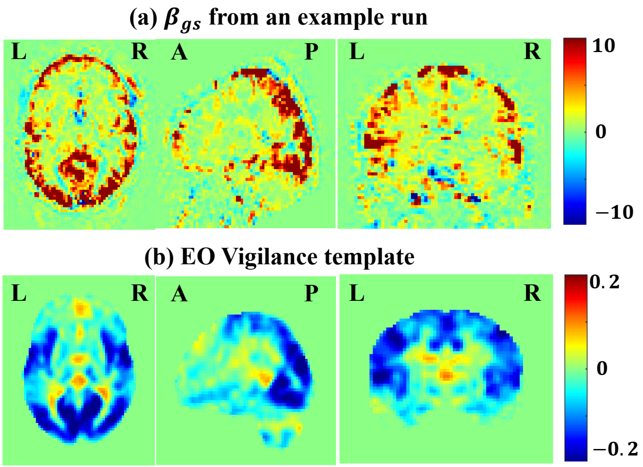

Figure S20: Visualization of (a) $\boldsymbol{\beta}_{\boldsymbol{g}_s}$ from the example run shown in Fig. 3 and (b) the eyes-open vigilance template estimated in (Falahpour et al., 2018). $\boldsymbol{\beta}_{\boldsymbol{g}_s}$ from the example run was calculated with the functional data after registration and transferring to MNI standard space. There is a strong negative correlation ($r = -0.38$, $p < 1 \times 10^{-6}$) between $\boldsymbol{\beta}_{\boldsymbol{g}_s}$ and the vigilance template.

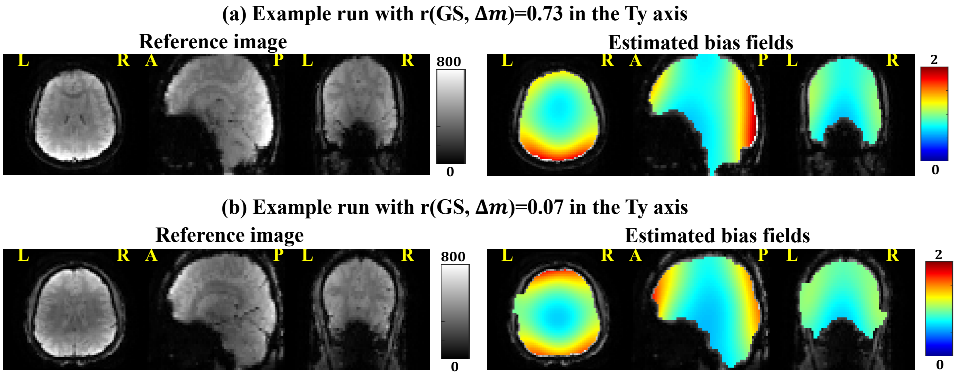

Figure S21: Visualization of the reference images and estimated bias fields from example runs with (a) high $r(\Delta\boldsymbol{m}, \mathrm{GS})$ and (b) low $r(\Delta\boldsymbol{m}, \mathrm{GS})$ in the Ty axis. The bias fields were estimated by SPM segmentation tool and were plotted within brain masks. Both the example runs are classified as low motion and high aGS. For the example run in (a), aGS $= 0.61$ and mean FD $= 0.07$mm. For the example run in (b), aGS $= 0.80$ and mean FD $= 0.05$mm.

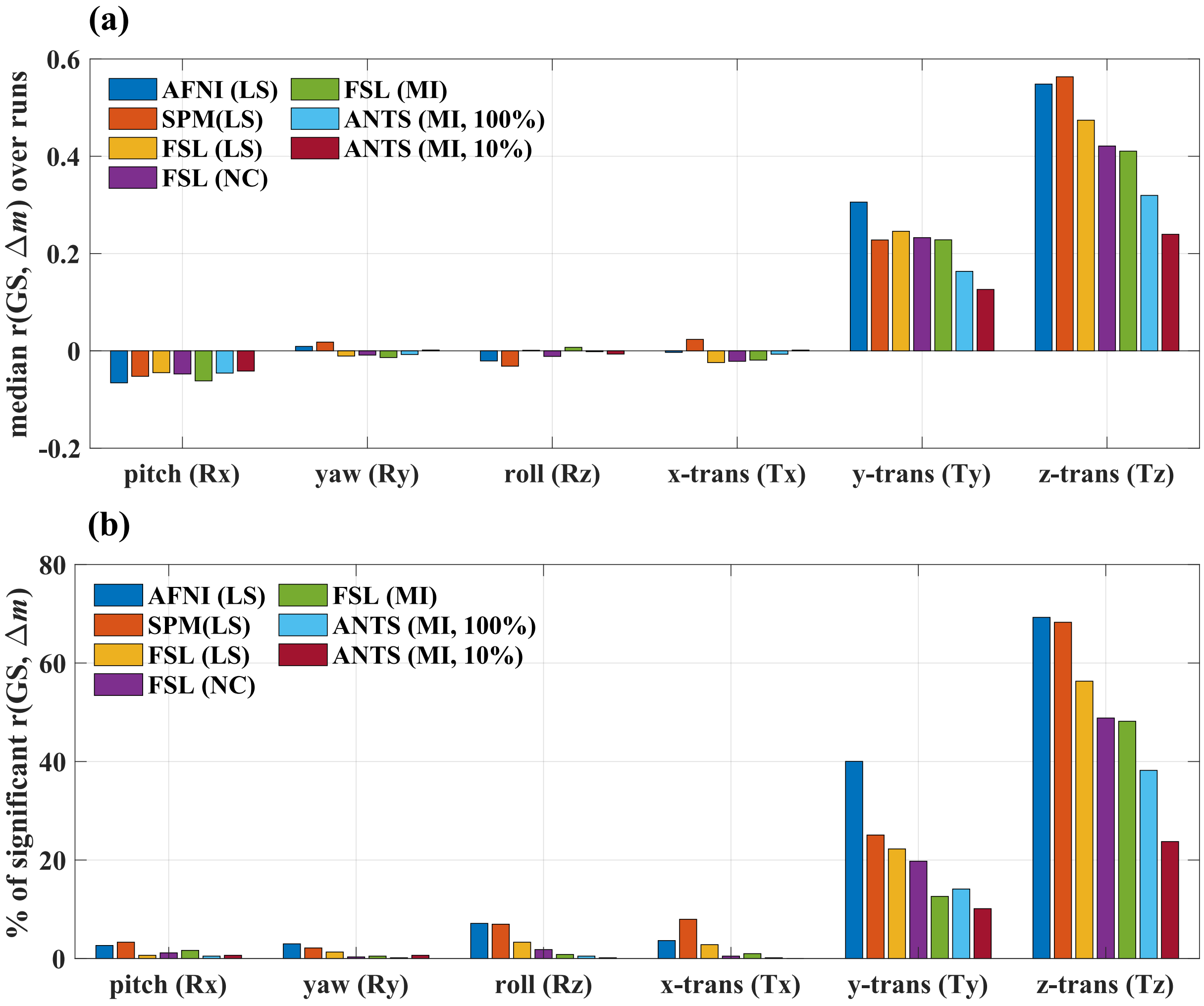

Figure S22: Comparisons of (a) mean $r(\Delta\boldsymbol{m}, \mathrm{GS})$ over runs and (b) the percentage of runs showing significant $r(\Delta\boldsymbol{m}, \mathrm{GS})$ values calculated with motion estimates from different algorithms (AFNI *3dvolreg*, SPM *spm_realign*, FSL *mcflirt*, ANTS *antsMotionCorr*) and cost functions (least-squares (LS), normalized correlation (NC), mutual information (MI)). For ANTS, the sampling percentage was set to either 10% or 100%. The $r(\Delta\boldsymbol{m}, \mathrm{GS})$ values were calculated after e1 motion regression. For each algorithm, cost function and motion axis, an empirical null distribution of $r(\Delta\boldsymbol{m}, \mathrm{GS})$ was generated by the permutation-based method and used to assess the significance of the measured $r(\Delta\boldsymbol{m}, \mathrm{GS})$ values. A Bonferroni-corrected run-wise p-value threshold of 0.05/602 was used, where 602 is the number of runs.

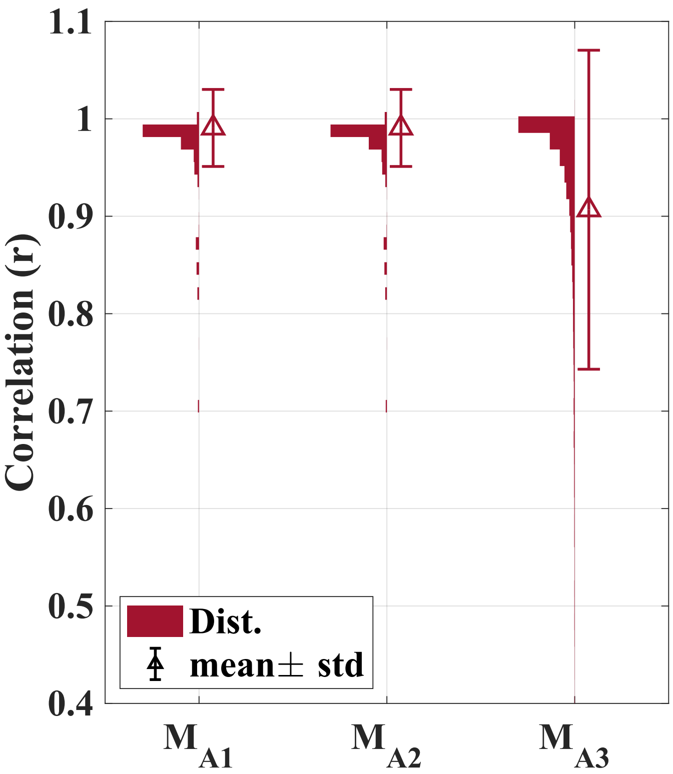

Figure S23: Distributions of the correlations between the motion estimates from AFNI *3dvolreg* and the approximated motion estimates, i.e. $\boldsymbol{M}_{Ai}$, $i = 1, 2$ and 3. The approximations are described in Eq. B.1. The distributions include the correlations for all six motion axes from all runs for both e1 and e2.

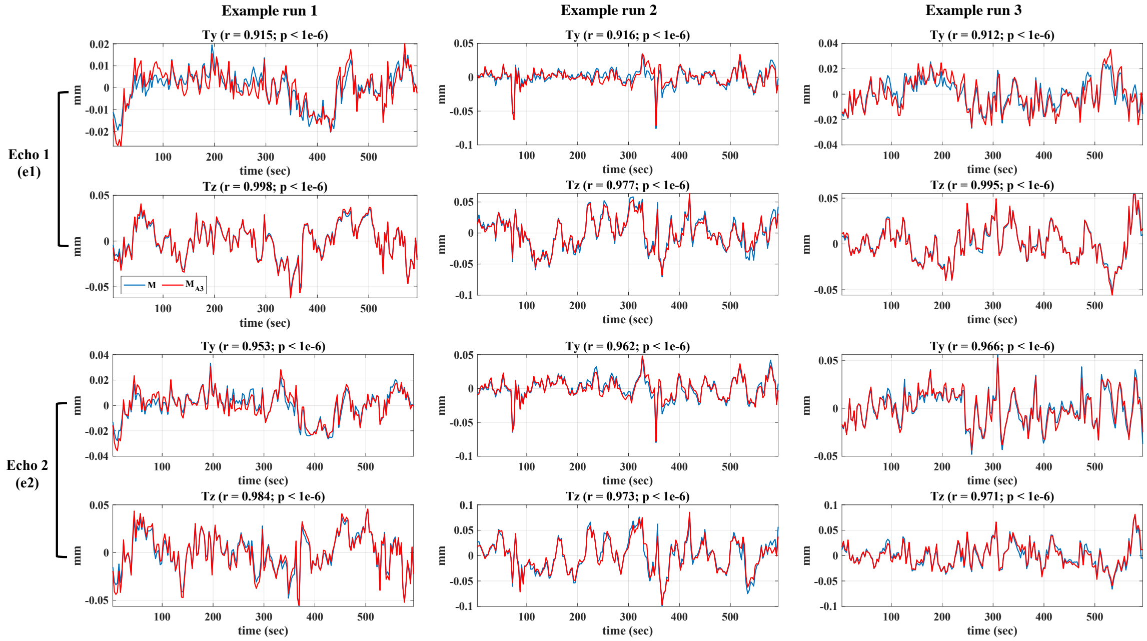

Figure S24: Comparison of the motion estimates from AFNI *3dvolreg* (i.e. $\boldsymbol{M}$, blue) and the motion estimates after the third approximation (i.e. $\boldsymbol{M}_{A3}$, red) in the Ty and Tz axes from three example runs. The top two rows show the motion estimates for e1, and the bottom two rows show the motion estimates for e2.

References

Avants, B.B., Tustison, N.J., Song, G., Cook, P.A., Klein, A., Gee, J.C., 2011. A reproducible evaluation of ANTs similarity metric performance in brain image registration. NeuroImage 54, 2033–2044. doi:10.1016/j.neuroimage.2010.09.025.

Avants, B.B., Tustison, N.J., Stauffer, M., Song, G., Wu, B., Gee, J.C., 2014. The Insight ToolKit image registration framework. Frontiers in Neuroinformatics 8, 44. doi:10.3389/fninf.2014.00044.

Belaroussi, B., Milles, J., Carme, S., Zhu, Y.M., Benoit-Cattin, H., 2006. Intensity non-uniformity correction in MRI: Existing methods and their validation. Medical Image Analysis 10, 234–246. doi:10.1016/j.media.2005.09.004.

Falahpour, M., Chang, C., Wong, C.W., Liu, T.T., 2018. Template-based prediction of vigilance fluctuations in resting-state fMRI. NeuroImage 174, 317–327. doi:10.1016/j.neuroimage.2018.03.012.

Falahpour, M., Wong, C.W., Liu, T.T., 2016. The resting state fMRI global signal is negatively correlated with time-varying EEG vigilance, in: Proceedings of the 24th Annual Meeting of the ISMRM, p. 641.

Freire, L., Mangin, J.F., 2001. Motion Correction Algorithms May Create Spurious Brain Activations in the Absence of Subject Motion. NeuroImage 14, 709–722. doi:10.1006/nimg.2001.0869.

Freire, L., Roche, A., Mangin, J.F., 2002. What is the Best Similarity Measure for Motion Correction in fMRI Time Series? IEEE Transactions on Medical Imaging 21, 470. doi:10.1109/tmi.2002.1009383.

Friston, K.J., Ashburner, J., Frith, C.D., Poline, J., Heather, J.D., Frackowiak, R.S.J., 1995. Spatial registration and normalization of images. Human Brain Mapping 3, 165–189. doi:10.1002/hbm.460030303.

Gispert, J.D., Reig, S., Pascau, J., Vaquero, J.J., García-Barreno, P., Desco, M., 2004. Method for bias field correction of brain T1-weighted magnetic resonance images minimizing segmentation error. Human Brain Mapping 22, 133–144. doi:10.1002/hbm.20013.

Gonzalez-Castillo, J., Duthie, K.N., Saad, Z.S., Chu, C., Bandettini, P.A., Luh, W.M., 2013. Effects of image contrast on functional MRI image registration. NeuroImage 67, 163–174. doi:10.1016/j.neuroimage.2012.10.076.

Jenkinson, M., Bannister, P., Brady, M., Smith, S., 2002. Improved Optimization for the Robust and Accurate Linear Registration and Motion Correction of Brain Images. NeuroImage 17, 825–841. doi:10.1006/nimg.2002.1132.

Jenkinson, M., Smith, S., 2001. A global optimisation method for robust affine registration of brain images. Medical Image Analysis 5, 143–156. doi:10.1016/s1361-8415(01)00036-6.

Laurita, A.C., DuPre, E., Ebner, N.C., Turner, G.R., Spreng, R.N., 2020. Default network interactivity during mentalizing about known others is modulated by age and social closeness. Social Cognitive and Affective Neuroscience 15, 537–549. doi:10.1093/scan/nsaa067.

Schaefer, A., Kong, R., Gordon, E.M., Laumann, T.O., Zuo, X.N., Holmes, A.J., Eickhoff, S.B., Yeo, B.T.T., 2018. Local-Global Parcellation of the Human Cerebral Cortex from Intrinsic Functional Connectivity MRI. Cerebral Cortex 28, 3095–3114. doi:10.1093/cercor/bhx179.

Setton, R., Mwilambwe-Tshilobo, L., Girn, M., Lockrow, A.W., Baracchini, G., Hughes, C., Lowe, A.J., Cassidy, B.N., Li, J., Luh, W.M., Bzdok, D., Leahy, R.M., Ge, T., Margulies, D.S., Misic, B., Bernhardt, B.C., Stevens, W.D., Brigard, F.D., Kundu, P., Turner, G.R., Spreng, R.N., 2022. Age differences in the functional architecture of the human brain. Cerebral Cortex 33, 114–134. doi:10.1093/cercor/bhac056.
